# Measuring the impact of monoclonal antibody therapies

**DOI:** 10.3389/fmed.2023.1256712

**Published:** 2023-11-15

**Authors:** Ka Keat Lim, Kenrick Ng, Sathiyaa Balachandran, Mark D. Russell, Amy Boalch, Alasdair Sinclair, Bolaji Coker, Krishna Vinnakota, Rashid Mansoor, Abdel Douiri, Lara V. Marks, Steven Sacks

**Affiliations:** ^1^Department of Population Health Sciences, King's College London, London, United Kingdom; ^2^Cancer Institute, University College London, London, United Kingdom; ^3^Centre for Rheumatic Diseases, King's College London, London, United Kingdom; ^4^University College London Hospitals NHS Foundation Trust, London, United Kingdom; ^5^NIHR Biomedical Research Centre, Guy's & St Thomas' NHS Foundation Trust, London, United Kingdom; ^6^University College London Hospitals NHS Foundation Trust (UCLH), London, United Kingdom; ^7^WhatisBiotechnology.org, Sevenoaks, United Kingdom; ^8^Peter Gorer Department of Immunology, King's College London, London, United Kingdom

**Keywords:** monoclonal antibodies, real world data (RWD), systematic literature review, meta-analysis, cost benefit evaluation

## Abstract

**Objective:**

Monoclonal antibody (Mab) treatments have significantly improved the quality and quantity of life, but they are some of the most expensive treatments, resulting in a degree of hesitancy to introduce new Mab agents. A system for estimating the effect of Mab drugs, in general, would optimally inform health strategy and fully realize how a single scientific discovery can deliver health benefits. We evaluated such a method with several well-established Mab regimens.

**Methods:**

We selected five different Mab regimens in oncology and rheumatology in England. We carried out two systematic literature reviews and meta-analyses to assess health outcomes (Health Assessment Questionnaire-Disability Index for rheumatoid arthritis; overall mortality for melanoma) from real-world data and compared them to the outcomes from randomized control trials (RCTs). We applied economic modeling to estimate the net monetary benefits for health outcomes for the estimated patient population size for each Mab regimen.

**Results:**

Meta-analyses of 27 eligible real-world data (RWD) sets and 26 randomized controlled trial (RCT) sets found close agreement between the observed and expected health outcomes. A Markov model showed the net positive monetary benefit in three Mab regimens and the negative benefit in two regimens. However, because of limited access to NHS data, the economic model made several assumptions about the number of treated patients and the cost of treatment to the NHS, the accuracy of which may affect the estimation of the net monetary benefit.

**Conclusion:**

RCT results reliably inform the real-world experience of Mab treatments. Calculation of the net monetary benefit by the algorithm described provides a valuable overall measure of the health impact, subject to the accuracy of data inputs. This study provides a compelling case for building a comprehensive, systematized, and accessible database and related analytics, on all Mab treatments within health services.

## Introduction

It has been nearly 50 years since Milstein and Kohler first described a method to produce monoclonal antibodies of desired specificity (1). The advancement/evolution with which this fundamental science translated into clinical medicine is historic. More than 100 Mab drugs have been licensed since the first one was approved in 1985. Twice as likely to succeed in clinical trials than small molecule drugs, Mab drugs currently account for nearly a fifth of the FDA's annual new drug approvals, with an average of ten approvals per year. Furthermore, the number of Mabs entering the clinic is rapidly increasing (2).

Few people outside scientific circles are aware of the impact of Mab drugs. For example, Mab drugs have transformed certain cancers from being labeled a terminal disease to a chronic condition (3, 4). They have also dramatically altered the treatment of autoimmune disorders such as rheumatoid arthritis (RA), moving the treatment away from merely relieving symptoms to better targeting and disrupting its pathogenesis (5, 6).

Many studies have examined the health benefits and cost effectiveness of individual Mab drugs (7–9), but there is a dearth of information on how many patients receive such drugs under the NHS and the extent to which their lives have benefited from them. Furthermore, little is known about how much the NHS pays for the drugs because they are secured based on an undisclosed discount with companies negotiated by individual NHS Trusts. This is important because Mabs are some of the most expensive drugs in the world. On average, they cost between $15,000 and $200,000 per patient per year (10). This reflects the complexity of producing therapeutic proteins compared to small-molecule drugs.

Just how much Mab drugs add to the NHS budget can be seen from the case of adalimumab in 2018. Prescribed to more than 46,000 patients for serious conditions such as RA, inflammatory bowel disease, and psoriasis, adalimumab was the biggest NHS spend that year (11). With the increasing use of Mab drugs, we believe it is time to understand more about the collective impact of Mab treatments and how this can be monitored in real time.

Our aim was to determine whether systematic literature review (SLR) combined with meta-analysis followed by economic modeling can assess the impact across a range of different Mab drug regimens in different clinical settings. We report the results of a pilot study on five well-established Mab regimens where the follow-up data are sufficiently mature for analysis. This includes immune-checkpoint inhibitors for melanoma that have radically improved life survival (4), and adalimumab treatment for RA (5, 6), the most prescribed biological agent for patients resistant to standard disease-modifying drugs (csDMARDs). Our results reflect the feasibility of assessing impact in this way, particularly the value of RCT at predicting health benefits in clinical practice and on the need for accessible real-world data to inform economic modeling.

## Methods

Considering that over fifty major conditions are currently treated with Mab drugs, we decided to focus this pilot study on two treatment groups that represent diverse conditions with proven benefits ranging from life-saving (melanoma) to life-enhancing (RA), each with enough cases to assess the real-world health and monetary impact. By selecting several Mab regimens for one of the conditions (melanoma), internal comparison was, in addition, possible.

Our study includes two SLRs for melanoma and RA, each with a meta-analysis to determine the health benefit of each drug regimen under consideration (Prospero registration CRD42021261882). The meta-analyses results, along with other data in the literature, were used to populate two economic models estimating the net monetary benefit for each Mab. The five Mab regimens analyzed are listed in Supplementary Table S1, together with their different approval dates.

### Identification, screening, and eligibility of mab studies for SLR

**Figure 1:**
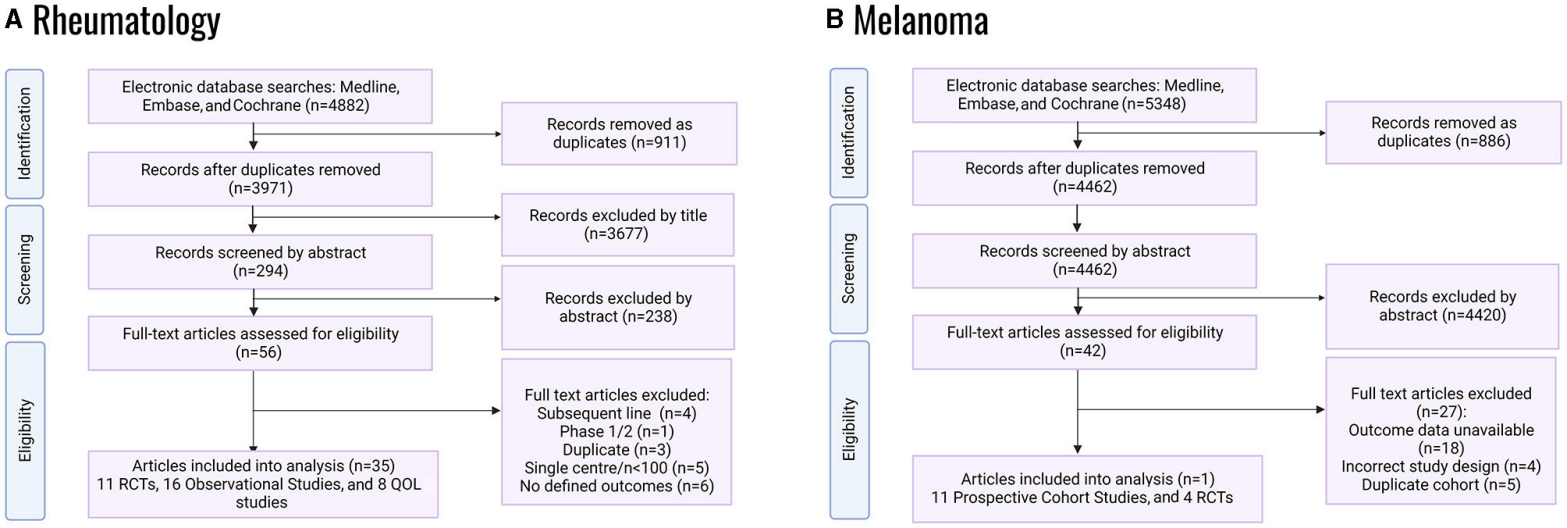
PRISMA flowcharts of systematic literature review for randomized control trials; QOL, quality of life: **(A)** RA studies, **(B)** melanoma studies.

Systematic database searches for each Mab regimen were conducted to identify randomized controlled trials (RCTs) and real-world data (RWD) studies. Figure 1 and Appendix 1 provide details of the databases searched. The databases identified were mainly phase II/III clinical trials, observational cohort studies, and post-market surveillance studies. Only patients aged 18 or older were included.

Melanoma studies were only eligible where all participants had a diagnosis of metastatic cutaneous melanoma or unresectable locally advanced melanoma. Participants needed to receive either pembrolizumab, ipilimumab or nivolumab, or the ipi-nivo combination as first-line treatments. The following studies were deemed ineligible: Phase I and non-randomized Phase II studies, studies with <100 participants, single-center studies, and studies that did not include treatment-naïve participants. The primary outcome for RCTs was progression-free survival (PFS); the secondary outcomes were median overall survival (OS) and quality of life index (EQ-5D or EORTC-QLQ-C30). For observational studies, the primary outcome was survival rate and the secondary outcome was hospitalization rate (proxied from Grade III and Grade IV toxicities).

Eligible RA studies included Phase III RCTs that compared adalimumab (40 mg subcutaneously every 2 weeks) with a placebo and captured data on Health Assessment Questionnaire-Disability Index outcomes (HAQ-DI, a measure of functional impairment) at drug initiation and 12-months post-initiation. Observational cohort studies were eligible where prospectively recruited adult RA patients were treated with adalimumab, and data on HAQ-DI outcomes at drug initiation and 12-months post-initiation were available. A comparator arm was non-essential for the inclusion of cohort studies. A stable background therapy with csDMARD was permissible for the inclusion in all studies. Extracted data included the following outcome measures at baseline and at 12- and 24-months post-adalimumab initiation: HAQ-DI Disease Activity Score-28 Joints (DAS28) and quality of life measures (EQ-5D, SF-36 and SF-12). The primary outcome measure was HAQ-DI at 12-months post-adalimumab initiation, relative to baseline. Secondary outcome measures were the change from baseline in HAQ-DI at 24-months post-adalimumab initiation; DAS-28 at 12- and 24-months post-adalimumab initiation; EQ-5D at 12- and 24-months post-adalimumab initiation; and SF-36/SF-12 at 12- and 24-months post-adalimumab initiation. For RCTs, comparison between the adalimumab and placebo study arms was made for the change from baseline in the above outcome measures.

### Statistical analysis

For melanoma, a random-effects model of network meta-analysis estimated the hazard ratios (HRs) with 95% confidence intervals (CIs) for OS reported in RCTs. The analyses were based on the reported HRs between trial arms.

For RA, random-effects meta-analyses of aggregated study data were conducted where more than two RCTs and observational cohort studies reported on outcome measures. The heterogeneity assessment used *I*^2^. Publication bias was assessed using funnel plots. Meta-analyses for RCTs and RWD studies were conducted separately. Sensitivity analysis was performed to exclude those studies where heterogeneity between studies was considered high and to assess the impact on effect-size estimates.

Statistical analysis used R (R Foundation for Statistical Computing, Vienna, Austria). A *p*-value lower than 0.05 was considered statistically significant.

### Estimating total and net monetary benefits on health outcomes of selected mab drugs

#### Model overview

To estimate the total and net monetary benefits of Mabs, we developed separate Markov models for melanoma and RA (Supplementary Figure S1). Markov models, common in economic evaluation, use health states to represent possible consequences of a health condition and allow patients to transition from one health state to another. The Markov model for melanoma comprised three health states: pre-progression, post-progression, and death (Supplementary Figure S1A), whereas the Markov model for RA comprised four health states: treated-with-methotrexate, treated-with-adalimumab, discontinued-from-adalimumab, and death (The Markov model, Supplementary Figure S1B). More details of the models are available as a footnote below Supplementary Figure S1. The Markov models accrue the cost and health outcomes [in quality-adjusted life years (QALYs)] of Mabs and their comparators for patients in England for lifetime (Supplementary Figure S1). Comparators were dacarbazine (historical standard of care cytotoxic treatment for melanoma) (12) and methotrexate (first-line treatment for RA).

The models required six input variables: (1) annual cost of Mab regimens and respective comparators; (2) utility value (UV) for each health state; (3) mortality; (4) probability of discontinuing Mab treatment; (5) patient population size in England; and (6) willingness to pay (WTP) (Appendix 2).

For melanoma, the mortality HRs for each Mab regimen relative to nivolumab was estimated by our SLR; the probability of death for nivolumab or the probabilities of transitioning to post-progression state for all Mabs were obtained from the follow-up of clinical trials. For RA, the probability of death followed that of England's life table, based on the assumption that RA has no effect on mortality. UVs were obtained from studies (13–15) identified through our SLR or studies (16, 17) identified in separate searches; in the treated-with-adalimumab-health state, UV was converted (Appendix 2; Equation 1) from HAQ-DI derived from our meta-analyses.

In base-case analyses, the total and net monetary benefits were estimated for each Mab using the base-case (mean) values of all data. A positive net monetary benefit would suggest that the Mab is potentially cost-effective at the specified level of WTP; a negative net monetary benefit suggests that the Mab may not be cost-effective.

The Mab regimen most recently recommended on July 2016 by NICE is ipi-nivo for melanoma (18). To compare Mab regimens over the same period, we accrued the QALYs and the costs over lifetime per patient and calculated total and net monetary benefits (Appendix 2, Equations 2–3) on QALYs for each Mab treatment per patient and for all patients over 4 years, 2017–2020.

One-way deterministic sensitivity analyses addressed whether the findings were sensitive to input variation, where the input value varied between the lower and upper limits, and the value of all other input variables was held constant. Included in the analyses were variations in the cost discount of Mabs (0-99%; arbitrary), WTP (£20,000–80,000 per QALY gained for melanoma; (19) £20,000–30,000 per QALY gained for RA) (20), and UVs (within 95% CIs). Also included in the analyses for melanoma were variation of HRs and probability of mortality (within 95% CIs). Threshold analyses addressed the values of the input variables at which the net monetary benefit would turn 0, i.e., the break-even value beyond which the conclusion on cost-effectiveness changes.

## Results

**Figure 2:**
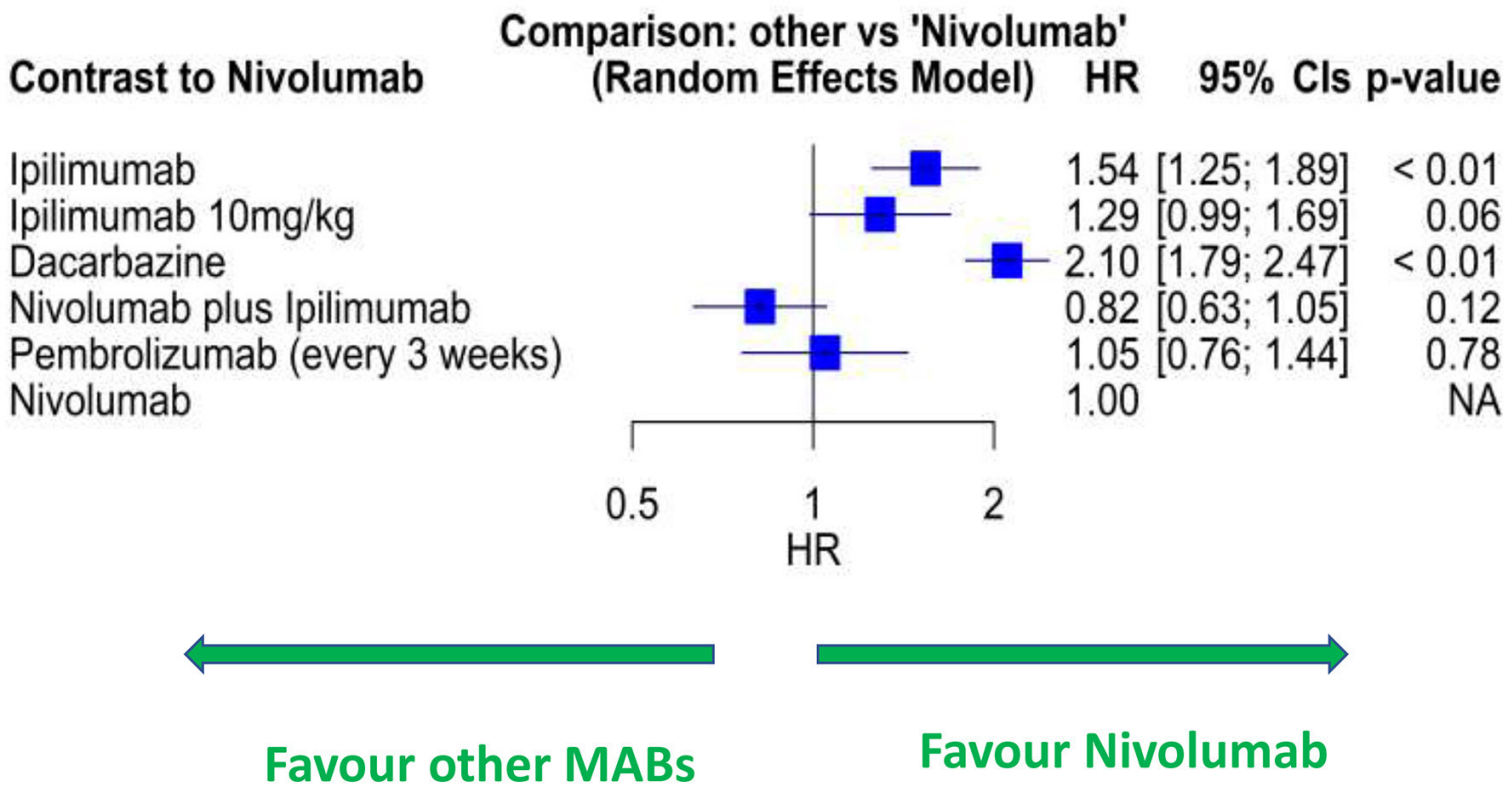
Forest plot of network meta-analysis of RCT data for selected Mab treatments for melanoma. HR, hazard ratio.

In the network meta-analysis of RCTs (Supplementary Figure S2), melanoma patients treated with ipi-nivo have a lower risk of dying than other Mab treatments (Figure 2; Supplementary Table S2). Compared to nivolumab, 3 mg/kg of ipilimumab was associated with 54% higher risk (HR: 1.54; 95% CIs: 1.25–1.89), 10 mg/kg of ipilimumab with 30% higher risk (HR: 1.29; 95% CIs: 0.99–1.69), and dacarbazine with a 2-fold higher risk (HR: 2.10; 95% CI: 1.79–2.47) of mortality. No differences were observed between nivolumab and pembrolizumab (HR: 1.05; 95% CIs: 0.76–1.44). However, ipi-nivo had 18% increased likelihood of survival compared with single-agent nivolumab (HR: 0.82; 95% CIs: 0.63–1.05). In the pooled analyses across RWD studies (Supplementary Figure S3; Supplementary Table S3), ipi-nivo had better OS than the treatment with single-agent anti-PD1 therapy (nivolumab or pembrolizumab) or ipilimumab; although these differences were not statistically significant, they agree with the RCT findings.

**Figure 3:**
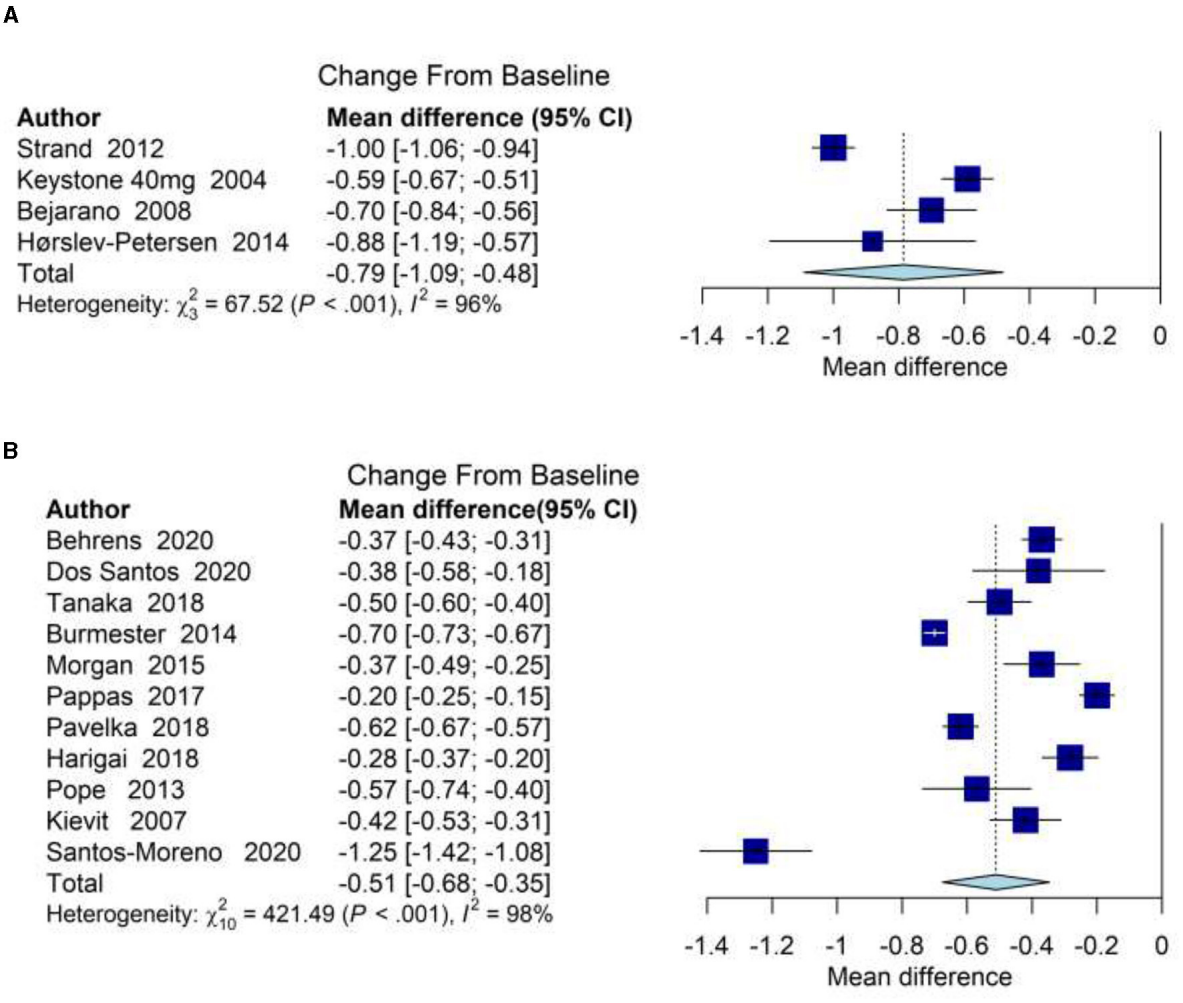
Forest plots displaying the weighted mean differences of HAQ-DI at 12 months, relative to baseline: **(A)** in RCTs of participants with RA treated with adalimumab; **(B)** in longitudinal cohorts of participants with RA treated with adalimumab.

The meta-analysis of health outcomes for RA patients treated with adalimumab confirmed improvements of function and disease-activity control with Mab therapy. Supplementary Tables S4–S6 and Supplementary Figures S4, S5 provide details on baseline characteristics, risk of bias, and outcome measures recorded in all studies. Across four eligible RCTs reporting HAQ-DI at baseline and 12 months, the weighted mean improvement in HAQ-DI was 0.79 (95% CIs: 0.48–1.09) at 12-month post-adalimumab initiation, relative to baseline (Figure 3A). Relative to placebo, adalimumab showed a mean improvement in HAQ-DI of 0.26 (95% CIs: 0.08–0.46) at 12 months (Supplementary Figure S6). In three eligible RCTs reporting data on DAS28 at baseline and 12 months, the weighted mean improvement in DAS28 was 2.81 (95% CIs: 2.15–3.47) at 12-month post-adalimumab initiation relative to baseline (Supplementary Figure S7). Relative to placebo, adalimumab-treated arms showed a mean improvement in DAS28 of 0.92 (95% CIs: 0.60–1.24) at 12 months (Supplementary Figure S8). Eligible RCTs reporting EQ-5D (*n* = 1) or SF-36 (*n* = 2) were too few to permit outcome analysis. Similarly, RCTs reporting HAQ-DI at 24 months (*n* = 1) or DAS28 at 24 months (*n* = 0) relative to baseline were too limited to permit outcome analysis. Sensitivity analysis after excluding studies from meta-analysis (based on the sample size and standard deviation) showed that our main conclusion was unaffected across the different analyses (Supplementary Figure S9).

In 11 eligible observational cohort studies of RA reporting HAQ-DI at baseline and 12 months, meta-analysis showed a weighted mean improvement in HAQ-DI of 0.51 (95% CIs: 0.35–0.68) at 12-month post-adalimumab initiation, relative to baseline (Figure 3B). Of nine eligible cohort studies reporting DAS28 at baseline and 12 months, the analysis showed a weighted mean improvement in DAS28 of 2.32 (95% CIs: 2.02–2.61) at 12 months relative to baseline (Supplementary Figure S10). Eligible cohort studies reporting EQ-5D (*n* = 1) or SF-36 (*n* = 2) were too few to permit meta-analysis of outcomes. Similarly, cohort studies reporting HAQ-DI at 24 months (*n* = 3) or DAS28 at 24 months (*n* = 3) relative to baseline were too scarce to permit meta-analysis of outcomes.

Clinical benefits of adalimumab treatment, measured by functional impairment and disease activity, were therefore evident both in real-world and trial settings (relative difference for HAQ-D1, 0.29 for DAS28, 0.49). HAQ-D1 and DAS28 were slightly higher at baseline in RCT than in RWD studies (Supplementary Figures S11–S14). Funnel plots of the change from baseline against standard error were symmetric, suggesting low risk of publication bias (Supplementary Figures S15, S16).

To determine the total and net monetary benefits on health outcomes for the five Mab regimens from approval to 2020, we estimated that 81,387 patients with RA and 2,324 patients with melanoma would have started their respective Mab regimens since they became available in the NHS (Supplementary Table S7). Of these, an estimated 33,255 had started adalimumab, 810 pembrolizumab, 405 ipi-nivo, 98 ipilimumab, and 37 nivolumab since 2017 (Supplementary Table S7).

Separately, we calculated lifetime QALYs and costs of Mabs accrued per patient (Supplementary Table S8). Adalimumab accrued the highest QALYs (16.33) per patient, followed by ipi-nivo (5.49), nivolumab (5.22), pembrolizumab (4.35), and lastly ipilimumab (3.21), all of which were higher than their respective comparators methotrexate (12.67) and dacarbazine (2.25). Ipi-nivo accrued the highest cost for Mabs, followed by pembrolizumab, ipilimumab, nivolumab, and adalimumab, ranging from £42,833 to £138,489, all higher than their comparators, methotrexate (£1,275) and dacarbazine (£2,433).

Using the total number of patients from 2017, lifetime QALYs, and costs per patient, we calculated the total and net monetary benefits to NHS England since their availability (Supplementary Table S8). The larger the number of patients treated, the higher the *total* monetary benefits—adalimumab £7.4 billion (*n* = 81,387 patients), pembrolizumab £91M (*n* = 827), ipi-nivo £71M (*n* = 438), ipilimumab £46M (*n* = 967), and nivolumab £7M (*n* = 47). Since 2017, total monetary benefits were smaller—adalimumab £3 billion (*n* = 33,255), pembrolizumab £84M (*n* = 810), ipi-nivo £65M (*n* = 405), nivolumab £55M (*n* = 37), and ipilimumab £47M (*n* = 98).

Accounting for the costs of Mabs, adalimumab generated a positive *net* monetary benefit of £1.8 billion (£1 billion from 2017), ipi-nivo generated £ 11M (£10M from 2017), and nivolumab generated £5M (£4M from 2017). However, pembrolizumab (–£10M) and ipilimumab (–£26M since its availability and –£3M from 2017) generated negative net monetary benefits. For adalimumab, while more *total* monetary benefits were accrued before biosimilars became available in the NHS in 2019, net monetary benefits were higher once biosimilars became available.

Sensitivity analyses determined how net monetary values varied at the lower and the upper limits of input variables (Supplementary Table S9; Appendix 3). Mabs had higher net monetary benefits at higher cost discounts, lower HRs of mortality (for ipilimumab, ipi-nivo, and pembrolizumab), lower probability of mortality (for nivolumab), higher UVs (baseline, change, or 1 year), and higher WTP.

Threshold analyses showed net monetary benefits for ipilimumab break-even with 35% discount from list price, and pembrolizumab at 11%, whereas ipi-nivo break-even at 20% cost increase and nivolumab at 3-fold cost increase (Table 1). The HR of mortality (vs. nivolumab) would need to be 1.32 for ipilimumab (vs. 1.54 at base-case), 1.00 for pembrolizumab (vs. 1.05 at base-case), and 0.90 for ipi-nivo (vs 0.82 at base-case) for the Mabs to break-even, baseline probability of mortality for nivolumab to be 0.18 (vs. 0.15 at base-case), baseline utility to be between 0.30 and 1.01 (vs. 0.65–0.80 at base-case, but utility >1 is implausible), change in UV to be between −0.43 and 0.18 (vs. −0.03 and 0.15 at base-case), or WTP to be ranging from £13,612 to 77,570 (vs. £50,000 at base-case).

**Table 1:** Base-case cost (£) of Mab treatments, net monetary benefit, and threshold cost at which the net monetary benefit was 0.

	**Base-case annual cost of mabs**	**Net monetary benefit at base-case annual cost (21)**	**Threshold cost for net return to turn 0**
**(a) Melanoma**			
Ipilimumab	75,000	−25,597,604	48,532 (35% discount)
Nivolumab	36,335	5,083,681	144,324 (297% increase)
Ipi-nivo	127,660	11,239,145	153,294 (20% increase)
Pembrolizumab (22)	118,525	−10,394,459	105,594 (11% discount)
**(b) Rheumatoid arthritis**			
Adalimumab (pre-2019)	9,156	3,033,193,847	14,469 (58% increase)
Adalimumab (post-2019)	3,679	3,033,193,847	27,166 (638% increase)

The table is reformatted from Supplementary Tables S4, S5.

Threshold analyses for adalimumab suggested the net monetary benefits would break-even at 58% cost increase from the list price (until 2018) or at 6.4-times cost increase from the reference price (since 2019), a HAQ-DI value of 1.36 (vs. 0.79 at base-case), UV of 0.54 for the discontinued-from-adalimumab state (vs. 0.62 at base-case), and 5.6% annual probability of discontinuing from adalimumab (vs. 10% at base-case), or at WTP of £ 16,241 per QALY gained (vs. £ 25,000 at base-case).

## Discussion

We have demonstrated that SLR combined with meta-analysis and subsequent health economic modeling can assess the impact across different Mab regimens used in different clinical contexts. Tested across five well-established Mab regimens in oncology and rheumatology where follow-up data were sufficiently mature for analysis, our data suggest the approach that may be suitable for applying more widely to other Mab regimens and indications. First, we observed a close agreement between the real-world health benefits and those anticipated from clinical trials. Second, we transformed these estimates of health benefits into a net monetary value for each Mab regimen by accounting for the perceived benefit over the comparator treatment, the number of patients receiving the treatment, actual drug costs, and WTP. While confirming the feasibility of this approach, the estimation of monetary return to the NHS depended on the accuracy of patient numbers and treatment costs for each Mab regimen, which presented a challenge to our analysis.

Few published studies have directly compared RWD sets with RCT sets for the same Mab regimens. Rigorously selecting a total of 27 eligible prospective cohort studies and 26 pivotal trial studies for side-by-side analysis, we were able to compare the health benefits perceived by patients in clinical trial and post-licensing settings. Our analysis confirmed that the health measures (HAQ-DI for RA and OS for melanoma) estimated in pivotal trials represented the benefit a patient could expect to receive following the introduction of the regimen into clinical practice. The slightly lower estimate of health measures for the RWD was perhaps because the group of patients included more advanced cases than in a trial setting. This observation is nonetheless important because it suggests that we are largely getting the health benefits predicted at the point national recommendations were made.

We developed a Markov model for each of the five regimens in melanoma and RA to accrue the lifetime QALYs and costs of Mabs and their comparators for all patients estimated to have received treatment since the Mabs became available in NHS England. In the five-year period from 2017, all Mabs accrued higher QALYs and costs per patient than their non-Mab comparators (ranging from £84M for pembrolizumab to £5M each for ipilimumab and nivolumab), with a total of £160M across the four Mab regimens. The net monetary benefits in England were positive for nivolumab and ipi-nivo but negative for pembrolizumab and ipilimumab because the lifetime cost of Mabs exceeded that of total monetary benefits. For RA, the additional QALYs from adalimumab compared to methotrexate translated to £3 billion (since 2017) and £12 billion net monetary benefit (since 2017), with the net benefit increasing since the availability of biosimilars in 2019.

Net monetary benefits increased with higher price discounts, HRs of mortality (melanoma), lower probability of mortality (melanoma), higher UVs (melanoma), lower HAQ-DI (RA), and a higher WTP per QALY. Threshold analyses revealed the values of input variables at which the Mabs would give 0 net monetary benefit, i.e., the values at which the conclusion on cost-effectiveness would change, although not all threshold values were plausible (e.g., UV > 1).

One important limitation was data access. We could not identify any appropriate data source for the actual patient-population size receiving the five Mab regimens in NHS England. Hence, we estimated the sizes based on reported incidence and prevalence data. However, we were unable to account for any fluctuations in the number of patients, e.g., adoption that may be slower early during its introduction or changes in patients diagnosed through the COVID pandemic. Furthermore, due to commercial confidentiality, we could not access the actual cost of Mabs borne by NHS England. Hence, the costs of Mabs in our estimations were based on list prices or reference prices, which we believe were higher than the actual cost. At list price, two of the five Mab regimens generated negative net monetary benefits; however, one-way sensitivity analyses revealed how the net monetary benefits would change with up to 10% discounts (Supplementary Table S9). We also conducted threshold analyses to identify the level of discounts at which the net monetary benefit would become 0. An example is pembrolizumab, where the net monetary value changed from a negative value to break even with a cost discount of 11% (Table 1). Because of this limitation of data access, the estimates of monetary benefit should be treated with caution; however, they are used here to model the calculation and sensitivity based on the data available at the time of the study.

Another limitation was the scope of the cost data. Our estimations only accounted for the cost of Mab drugs and their administration. This assumes that the antibodies and their comparators do not differ in their downstream health-service usage, which may be plausible for melanoma due to its high mortality rate but less plausible for RA because it affects patients' quality of life and not just their mortality. This, coupled with the accrual of lifetime QALYs, suggests that the net monetary benefits are ceiling estimates. Our findings may also be sensitive to the comparators, both of which cost less than the Mab regimens.

The fact that each drug was introduced at different times (see Supplementary Table S1) also poses a challenge in terms of the ability to directly compare the cost of the drugs. This is because their adoption, frequency of administration, and cost will have varied over time. Another important factor to consider is the patent expiry of each drug, which enables the production of biosimilars, that is, alternative products that demonstrate the same safety, efficacy, and quality of the original biological medicine but are substantially less expensive. At present, the only drug in this study for which there are biosimilars is adalimumab. Just how much savings biosimilars can offer can be seen from the fact that, in November 2018, NHS England estimated that it would save £300 million a year by switching to a biosimilar version of adalimumab (23). Within 6 months, nearly 63% of NHS patients switched over to such a biosimilar after their introduction (24).

Other biosimilars are likely to be introduced in the future, which will have a bearing on the other drugs for melanoma in this study, which going forward will need to be factored into the treatment algorithms for that condition. New regulatory guidance issued by the UK's Medicine and Healthcare Regulatory Agency in 2021 will help accelerate the licensing of new biosimilars in the UK (25). Patents for all Mab drugs for melanoma investigated in this study are due to expire toward the end of the 2020s.

Despite the limitations, our results have important implications for research practice and policy. Our results highlight the need for systematic data collection to assess the impact (in terms of total and net monetary benefits) of innovative treatments, such as Mabs on the NHS, particularly the number of treated patients and Mab drug costs. Prospective accrual of data from the time a Mab treatment is approved would avoid reliance on sporadic, retrospective, and incomplete dataset analysis. Our difficulty in accessing data during the current study strongly supports the case for an NHS-based system of data collection and analytics capturing all Mab treatments. Users would then derive the same benefits as for other comprehensive databases, e.g., the organ transplant database, which facilitates ongoing comparison of different practices across the NHS using standardized metrics to assess quality of life and transplant survival. Meaningful estimates of return of research investment would also incentivise publicly funded research on innovative (though costly) biological treatments such as Mab drugs.

## *Panel*: research in context

### Evidence before this study

Multiple studies and meta-analyses have examined the utility of individual Mab drugs approved for treatment in the NHS. Few studies have considered the overall health and economic benefits of this genre of drugs, as well as how it compares to the predicted benefits from pivotal clinical trials.

### Added value of this study

We show the relationship between clinical trials and real-world data on the outcome of different Mab treatments and diseases. Based on this information, we calculate the economic benefit to the NHS based on actual patient numbers. We illustrate how difficulties obtaining accurate information on patient numbers and Mab drug costs can distort the calculation of overall benefit.

### Implications of all the available evidence

With an ever-increasing number of diseases benefiting from Mab therapies, our study emphasises the need for a systematic and comprehensive scheme for accurate data collection and analytics within the NHS. This will be useful to benchmark the overall value of Mab drugs and inform health strategy and equitable access. It is also a celebration of the underpinning science as it approaches its 50th year since first reported.

## Data availability statement

The original contributions presented in the study are included in the article/Supplementary material, further inquiries can be directed to the corresponding author.

## Author contributions

KL: Conceptualization, Formal analysis, Investigation, Methodology, Writing—original draft. KN: Formal analysis, Methodology, Writing—original draft, Conceptualization, Data curation. SB: Data curation, Writing—original draft. MR: Conceptualization, Data curation, Formal analysis, Methodology, Writing—original draft. AB: Data curation, Writing—review & editing. AS: Data curation, Writing—review & editing. BC: Data curation, Writing—review & editing. KV: Data curation, Writing—review & editing. RM: Conceptualization, Data curation, Formal analysis, Methodology, Writing—original draft. AD: Conceptualization, Data curation, Formal analysis, Methodology, Writing—original draft. LM: Conceptualization, Funding acquisition, Project administration, Writing—original draft. SS: Conceptualization, Formal analysis, Funding acquisition, Methodology, Writing—original draft.
