# Supplementary material for: Measuring the impact of monoclonal antibody therapies
Source: Front Med (Lausanne). 2023 Nov 15;10:1256712. doi: 10.3389/fmed.2023.1256712 (PMC10690588; doi:10.3389/fmed.2023.1256712)
Supplement: Supplementary file 1 [file Data_Sheet_1.pdf]

Supplementary material

Table of contents

Supplementary figures

Fig S1 Markov model diagram for melanoma (A) and rheumatoid arthritis (B)

Fig S2. Network plot from the network meta-analysis for melanoma

Fig S2. Forest plot of median overall survival across melanoma drugs

Fig S4. Risk of bias assessment of eligible RCTs of RA participants, assessed using Cochrane Risk of Bias 2 (ROB2) tool

Fig S5: Risk of bias assessment of eligible prospective cohort studies of RA participants, assessed using the Newcastle Ottawa Score (NOS)

Fig S6. Summary of HAQ outcomes in RCTs, comparing adalimumab and placebo study arms at 12 months, relative to baseline

Fig S7. Summary of DAS28 outcome in RCTs, showing the change from baseline to 12 months in adalimumab-treated arms

Fig S8. Summary of DAS28 outcomes in RCTs, comparing adalimumab and placebo study arms at 12 months, relative to baseline

Fig S9. Sensitivity Analysis: Forest plot displaying the weighted mean differences in HAQ-DI at 12 months, relative to baseline in longitudinal cohorts of participants with RA treated with adalimumab, following exclusion of heterogeneous studies

Fig S10. Summary of DAS28 outcomes from real world studies, showing the change from baseline to 12 months in adalimumab-treated patients

Fig S11. HAQ-DI at baseline in adalimumab-treated participants of eligible RCTs

Fig S12. HAQ-DI at baseline in adalimumab-treated participants of eligible real-world studies

Fig S13. DAS28 at baseline in adalimumab-treated participants of eligible RCTs

Fig S14. DAS28 at baseline in adalimumab-treated participants of eligible real-world studies

Fig S15. Funnel plot for the HAQ outcome in RCTs, comparing adalimumab and placebo change from baseline to 12 months

Fig S16. Funnel plot for the DAS28 outcome in RCTs, comparing adalimumab and placebo at follow-up (12 months)

Supplementary tables

Table S1. Mab drug regimens selected for meta-analysis

Table S2. Baseline characteristics of the 4 eligible RCTs of melanoma patients

Table S3. Baseline characteristics of the 15 eligible longitudinal cohort studies of melanoma patients

Table S4. Baseline characteristics of the 4 eligible RCTs of RA patients

Table S5. Baseline characteristics of the 11 eligible longitudinal cohort studies of RA patients

Table S6. Summary table of outcome measures recorded in selected RCTs and longitudinal cohort studies of RA patients treated with adalimumab

Table S7. Estimated number of new patients starting treatment since the Mabs became available in NHS England until 2020

Table S8. Estimated lifetime QALYs, cost (£) of Mabs, total patients, total monetary benefit, and net monetary benefit, in NHS England

Table S9. One-way sensitivity analyses to examine how the net monetary values (£) changed with the lower and the upper limits of input variables, with the threshold values at which the net monetary benefit turned 0

Appendices

Appendix 1. Database details

Appendix 2. Summary of data sources for input variables for the Markov models

Appendix 3. Input variables point estimates and ranges

Appendix 4. Calculations of patient population size in England for (A) Stages 3 or 4 unresectable melanoma and (B) RA

Appendix 5. Dose, frequency, and duration of administration assumed in calculating the cost of the Mabs

Appendix 6. List of historical data sources explored for population size

Supplemental figures

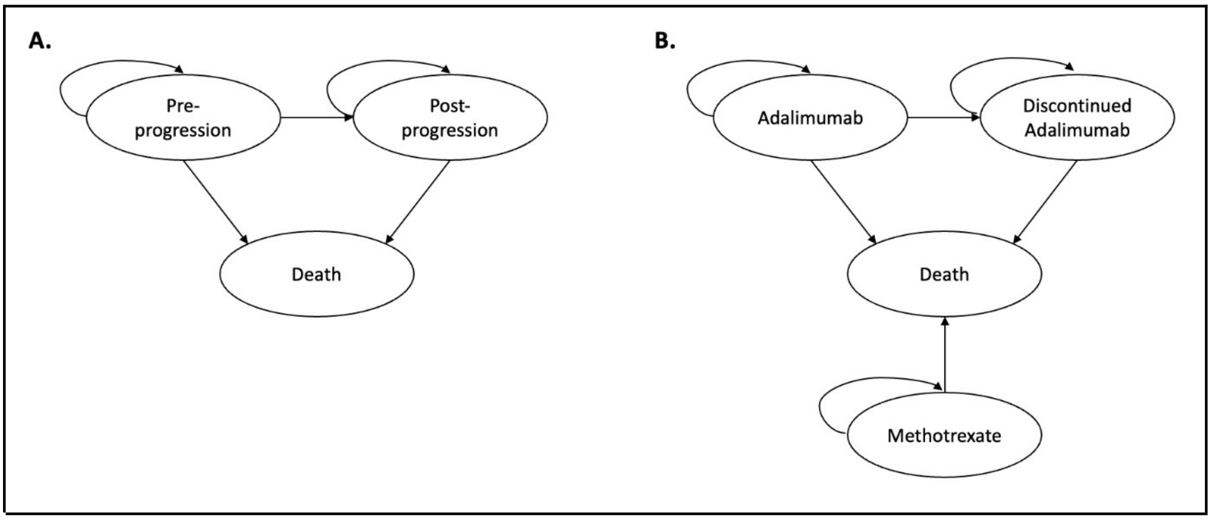

Figure S1. Markov model diagram for melanoma (A) and rheumatoid arthritis (B). (A) one course of Mabs is given in the pre-progression phase. (B) assumes patients take the medication for a patient's lifetime.

The Markov model for melanoma comprised three health states: pre-progression; post-progression; and death (Fig S1A). Patients may transition from pre-progression to post-progression, but not back as patients who have clinically progressed will not return to the pre-progression state. Patients at the pre-progression or post-progression state may also remain within the same health state (curved arrows) or transition to death. The transition probabilities between the health states differed between regimens. The three-state model structure is common in published literature[1] and reflects the changes in health status among patients with stages 3 or 4 melanoma. Patients transition between the health states in annual cycles, and they accrue costs and QALYs over their lifetime.

The Markov model for RA comprised four health states: treated-with-methotrexate, treated-with-adalimumab; discontinued-from-adalimumab; and death (Fig S1B). Patients taking methotrexate may transition from treated-with-methotrexate to death, whereas patients taking adalimumab may transition from treated-with-adalimumab to death or discontinued-from-adalimumab, but not the converse because patients who discontinue adalimumab usually do so due to non- or poor-response to ('failed') adalimumab. The four-state model structure is simplified from published models[2] that usually describe patients transitioning across successive treatments after failing the previous ones.

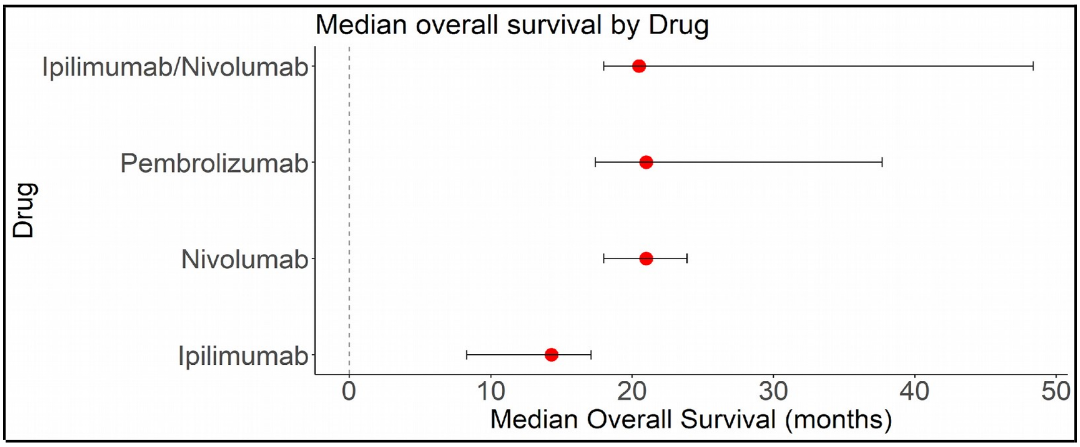

Figure S2. Forest plot of median overall survival across melanoma drugs

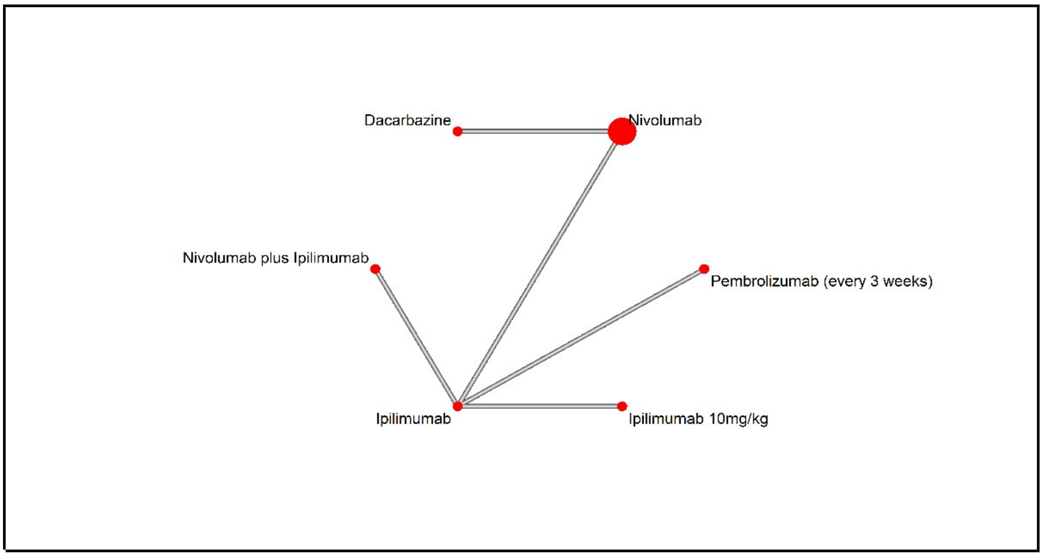

Figure S3. Network plot from the network meta-analysis for melanoma

Study ID	D1	D2	D3	D4	D5	Overall
Bejarano	+	!	!	+	+	!
Keystone	+	+	+	+	+	+
Hørslev-Petersen	+	+	+	+	+	+
Strand	+	+	+	+	+	+

+ Low Risk

! Some Concern

- High Risk

D1 - Randomisation process

D2 - Deviations from intended interventions

D3 - Missing outcome data

D4 - Measurement of the outcome

D5 - Selection of reported result

Figure S4. Risk of bias assessment of eligible RCTs of RA participants, assessed using Cochrane Risk of Bias 2 (ROB2) tool.[3] Study ID:[4, 5, 6, 7]

Author	Selection				Comparability		Outcome			Overall Score (8)
	1	2	3	4	A	B	1	2	3	
Behrens	*	N/A	*	*	*	*	*	*		7
Dos Santos	*	N/A	*	*		*	*	*		6
Tanaka	*	N/A	*	*	*	*	*	*		7
Santos-Moreno	*	N/A	*	*			*	*	*	6
Pope	*	N/A		*	*	*	*	*		6
Burmester	*	N/A	*	*			*	*	*	6
Kievit	*	N/A	*	*	*	*	*	*	*	8
Morgan	*	N/A	*	*	*	*		*	*	7
Pappas	*	N/A	*	*			*	*		5
Harigai	*	N/A	*	*	*	*	*	*		7
Pavelka	*	N/A	*	*			*	*		5

Figure S5: Risk of bias assessment of eligible prospective cohort studies of RA participants, assessed using the Newcastle Ottawa Score (NOS) The maximum possible score for a study is eight, as column two in the selection category did not apply to all studies.[8] Authors: [9, 10, 11, 12, 13, 14, 15, 16, 17, 18, 19]

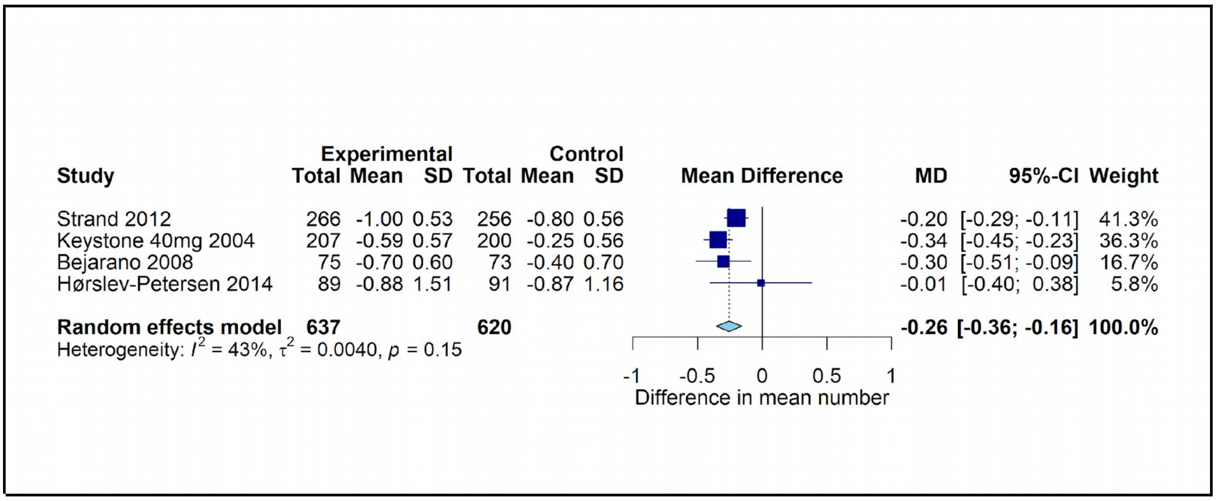

Figure S6. Summary of HAQ outcomes in RCTs, comparing adalimumab and placebo study arms at 12 months, relative to baseline. Studies:[4, 5, 6, 7]

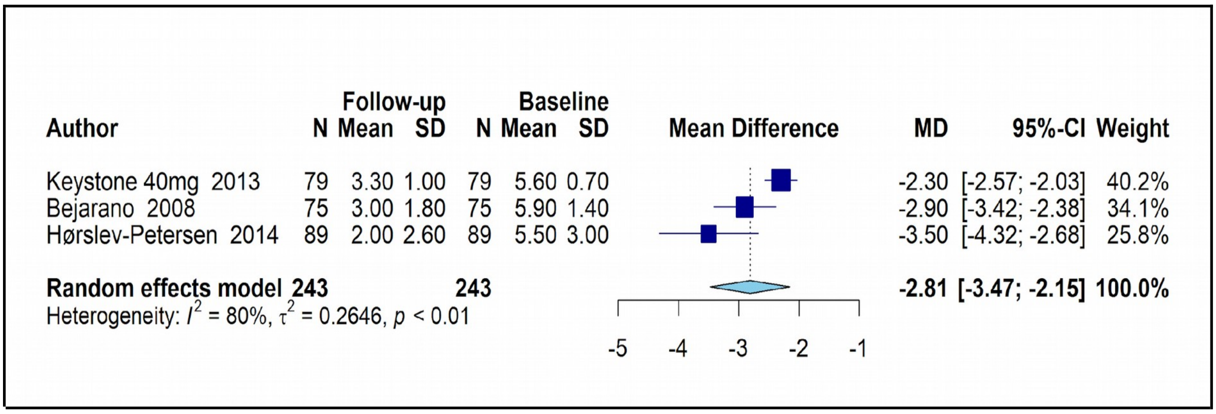

Figure S7. Summary of DAS28 outcome in RCTs, showing the change from baseline to 12 months in adalimumab-treated arms. Authors:[4, 5, 6]

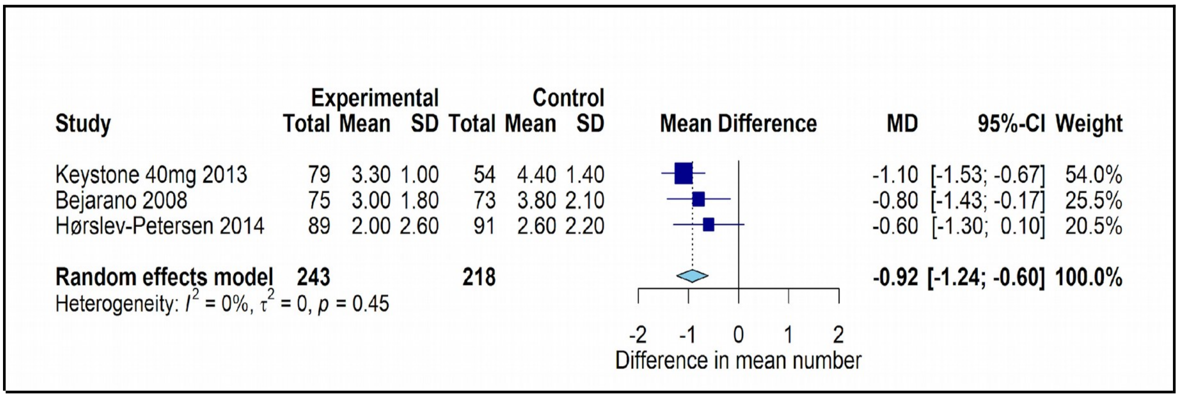

Figure S8. Summary of DAS28 outcomes in RCTs, comparing adalimumab and placebo study arms at 12 months, relative to baseline. Authors:[4, 5, 6]

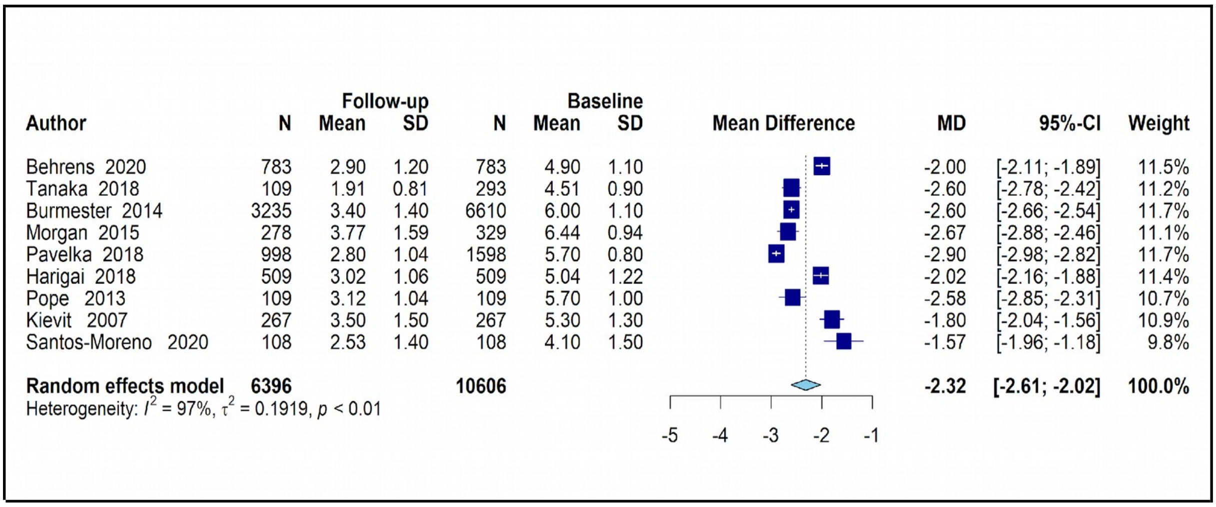

Figure S9. Summary of DAS28 outcomes from real world studies, showing the change from baseline to 12 months in adalimumab-treated patients. Authors:[9, 10, 12, 13, 14, 16, 17, 18, 19]

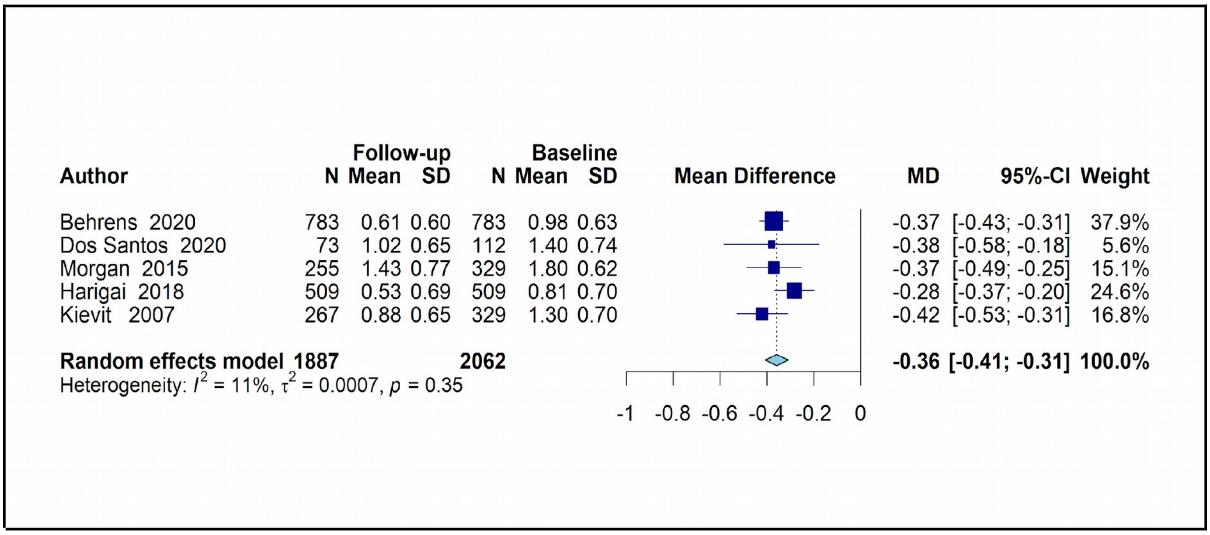

Figure S10. Sensitivity Analysis: Forest plot displaying the weighted mean differences in HAQ-DI at 12 months, relative to baseline in longitudinal cohorts of participants with RA treated with adalimumab, following exclusion of heterogeneous studies. Authors:[9, 11, 12, 13, 14]

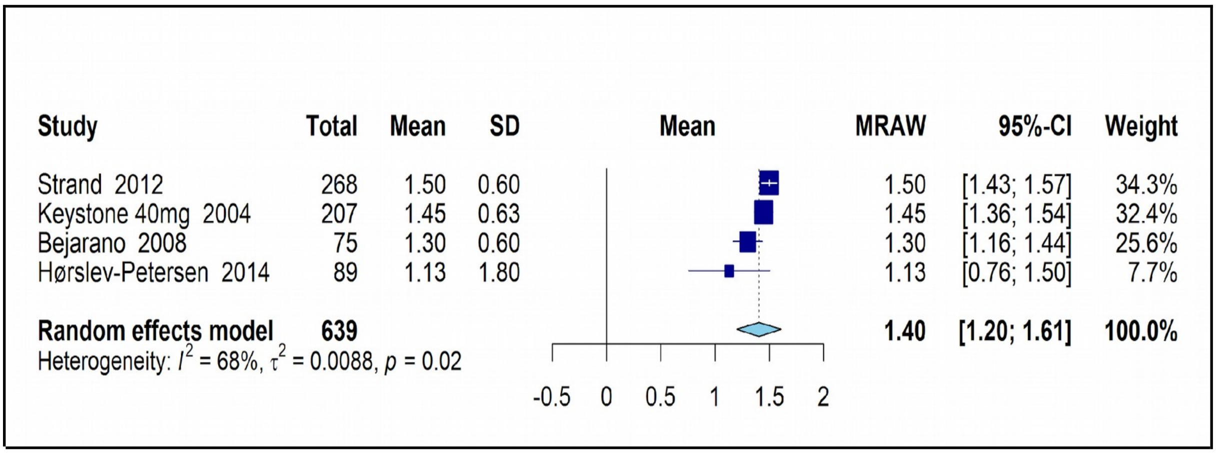

Figure S11. HAQ-DI at baseline in adalimumab-treated participants of eligible RCTs. Authors:[4, 5, 6, 7]

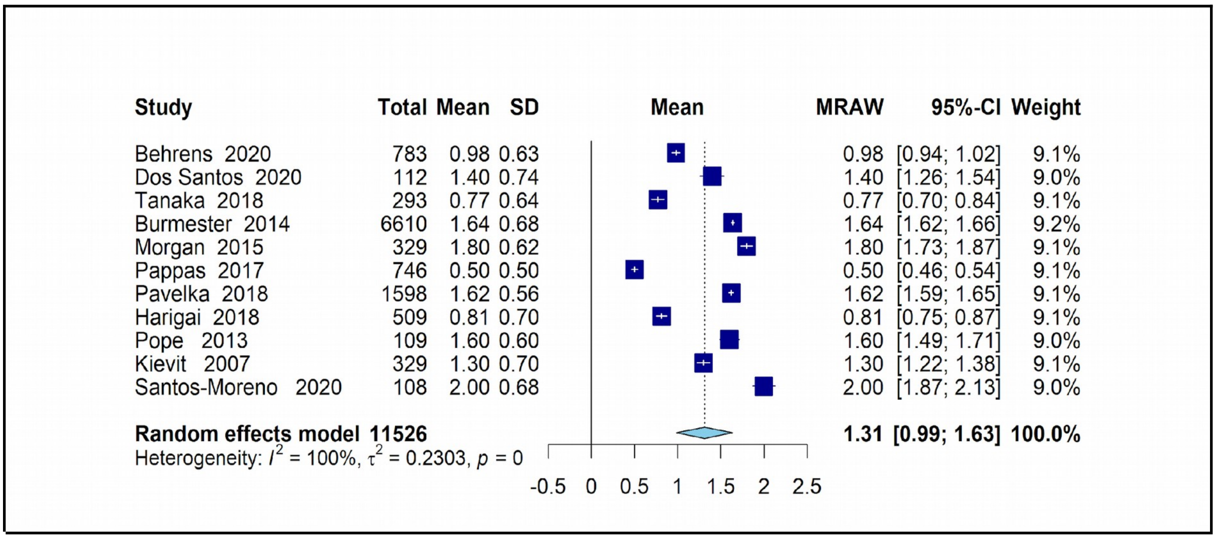

Figure S12. HAQ-DI at baseline in adalimumab-treated participants of eligible real-world studies. Studies:[9, 10, 11, 12, 13, 14, 15, 16, 17, 18, 19]

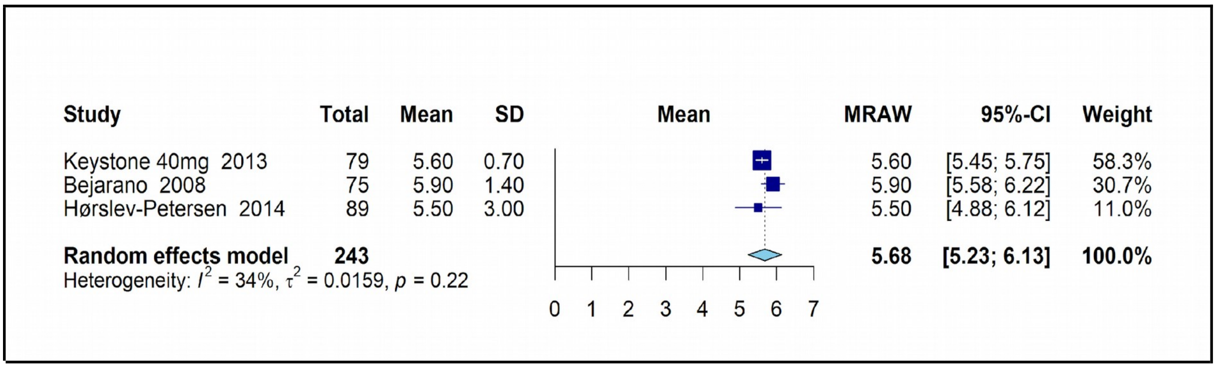

Figure S13. DAS28 at baseline in adalimumab-treated participants of eligible RCTs. Studies:[4, 5, 6]

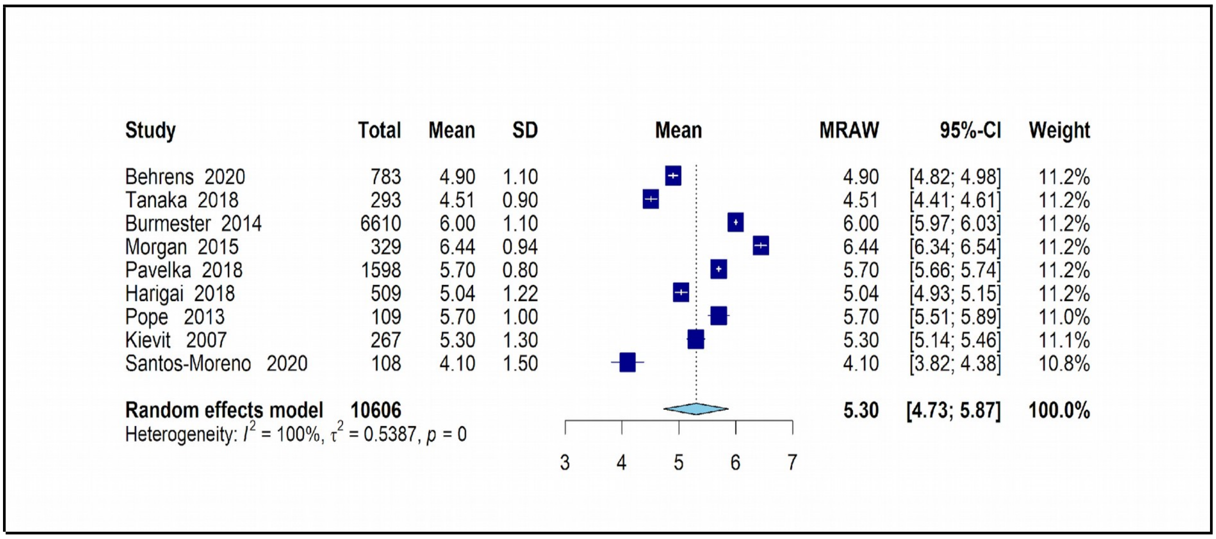

Figure S14. DAS28 at baseline in adalimumab-treated participants of eligible real-world studies. Studies:[9, 10, 12, 13, 14, 16, 17, 18, 19]

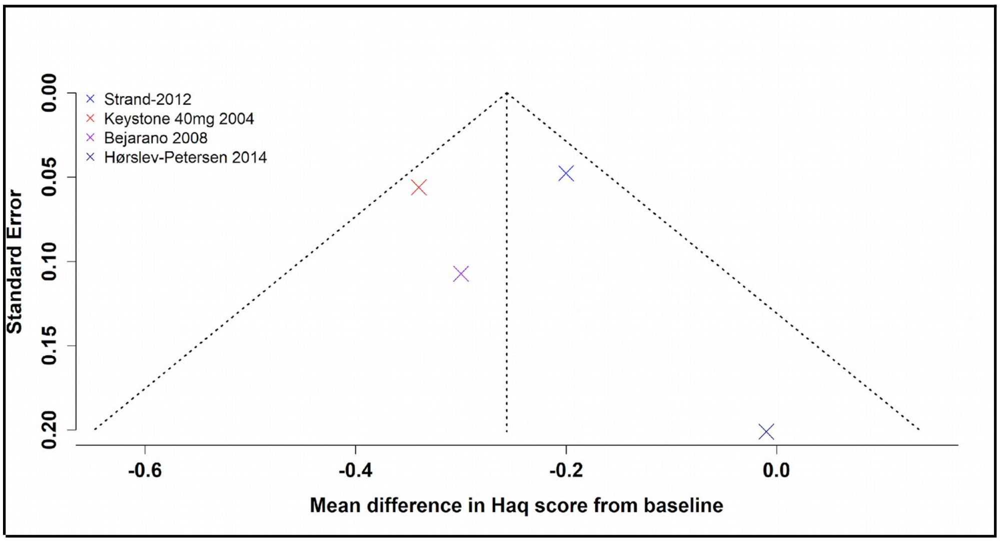

Figure S15. Funnel plot for the HAQ outcome in RCTs, comparing adalimumab and placebo change from baseline to 12 months. Authors:[4, 5, 6, 7]

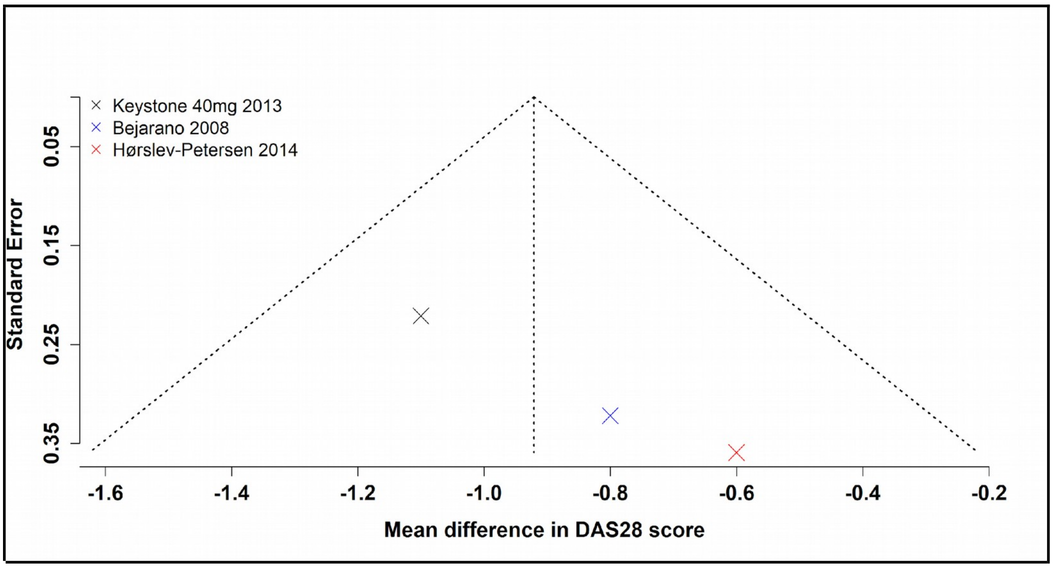

Figure S16. Funnel plot for the DAS28 outcome in RCTs, comparing adalimumab and placebo at follow-up (12 months). Authors:[4, 5, 6]

Supplementary tables

Mab name	Target	Indication	Year approved for use in UK
Ipilimumab	CTLA-4*	Melanoma	2011
Pembrolizumab	PD-1**	Melanoma	2014
Nivolumab	PD-1	Melanoma	2015
Ipi-nivo***	CTLA-4/PD-1	Melanoma	2016
Adalimumab	TNF-alpha	Rheumatoid arthritis	2003

Table S1. Mab drug regimens selected for meta-analysis

*CTLA-4, cytotoxic T lymphocyte associated antigen 4; **PD1, programmed cell death 1; ***Ipilimumab-nivolumab

The melanoma Mab treatments have enabled patients with unresectable Stage III melanoma and Stage IV melanoma to achieve 5-year overall survival rates of 40-50%[20, 21] compared to 6-9 months for the previous standard of care with dacarbazine.[22] Licensed indications have since extended to other cancers such as renal cell carcinoma, colorectal carcinoma, lung cancer, head and neck cancer, cervical cancer, Hodgkin lymphoma and certain types of breast cancer. Rheumatoid arthritis treatment with adalimumab, which inhibits tumour necrosis factor alpha, is given to patients who have failed to adequately respond to conventional synthetic disease-modifying antirheumatic drugs (csDMARDs). It produces significant reductions in pain, swelling and joint damage.[23, 24] Clinical indications also include other forms of autoimmune inflammatory arthritis (e.g., axial spondyloarthritis, psoriatic arthritis), inflammatory bowel disease, and psoriasis.

Author, year	Number of patients	Study drugs	Line of treatment, 1st, 2nd	Median FU (mo.)	Age range	Sex ratio (% F)	PS 0-1 (%)	BRAF Wt. (%)
Schachter 2017	834	P, three-weekly	1,2	22.9	22-89	37.2	100	65
Larkin 2019	945	IN	1	54.6	18-86	34	99	68
Ascierto 2020	831	I, 3mg/kg	1	43	IQR: 51-71	36	100	79
Robert 2020	418	N	1	60	18-87	41.1	99.5	100

Table S2. Baseline characteristics of the four eligible RCTs of Melanoma patients. I, Ipilimumab; N, Nivolumab; IN, Ipi-nivo; P, Pembrolizumab. Authors:[21, 25, 26, 27]

Author, year	Country	Number of patients	Study drugs	Line of treatment 1st, 2nd, 3rd	Median FU (mo.)	Age Range	Sex ratio (% F)	PS 0-1 (%)	BRAF Wt. (%)
Margolin, 2015	USA	273	I	1	12.2	26-91	35.2	80.6	66.3
Cowey, 2018	USA	168	P	1,2,3	10.5	26-90	35	64	65
Jochems, 2018	Nether-lands	807	I	1,2	11.5	41-79	38	88	60
Mohr , 2018	Inter-national	371	I	1,2,3	10.5	22-88	38	94	61
Tarhini, 2019	USA	487	I,N,IN	1	14	NA	45	86	75
Moser, 2019	USA	567	I	1	22.4	49-65	NA	56.5	0
Liu, 2019	USA	532	P	1,2,3	13.6	18-84	32.1	80	62
Hogg, 2020	Canada	194	I	1	12.9	27-81	35	100	51
Borges, 2021	Portugal	125	P	1,2,3	16.9	37-91	48.8	75	62.4
Moser, 2020	USA	888	P, N	1	17.3	IQR: 66-82	32	81	76
Pavlick, 2021	USA	557	IN	1	15.9	NA	35	79	0
Board, 2018	England	2322	P, I, N, IN	1	18	17-97	45	75	NA
Cowey, 2021	USA	303	P	1,2,3	18.2	26-90	34	74	75
Dalle, 2021	International	1356	I	1	36	22-90	40	NA	60
Casarotto, 2021	France	223	P	1,2,3	25.3	24-90	51	94	84

Table S3. Baseline characteristics of the 15 eligible longitudinal cohort studies of melanoma patients. I, **Ipilimumab; N, Nivolumab; IN, Ipinivo; P, Pembrolizumab.**

Authors:[28, 29, 30, 31, 32, 33, 34, 35, 36, 37, 38, 39, 40, 41, 42]

Author, year	Country	Number of Participants	Mean Age (SD)	Sex (% Male)	Background Therapy	Comparator	Outcomes reported
Keystone, 2004	N. America	207	56.1 (13.5)	23.7	MTX	Placebo	HAQ 12 mo
Bejarano, 2008	UK	75	47 (9)	41.6	MTX	Placebo	HAQ 12 mo<br>DAS28 12 mo
Strand, 2021	N. America, Europe, Australia	266	51.9 (14)	28	MTX	Placebo	HAQ 12/24 mo<br>SF36 12/24 mo
Horslev-Peterson, 2014	Denmark	89	56.3*	37	MTX	Placebo	HAQ 12 mo<br>DAS28 12 mo<br>SF12 12 mo<br>EQ5D 12 mo

Table S4. Baseline characteristics of the four eligible RCTs of RA patients *Median age. Authors:[4, 5, 6, 7]

Author, year	Country	Number of Participants	Mean Age (SD)	Sex (% Male)	Background Therapy	Outcomes Reported
Kievit, 2007	Netherlands	267	55.1 (12.6)	30	csDMARDs	HAQ 12 mo<br>DAS28 12 mo
Pope, 2013	Canada	109	56 (12.9)	16.5		HAQ 12 mo<br>DAS28 12 mo
Burmester, 2014	Europe	3235	53.7 (13)	19.3	MTX, LEF, SSZ	HAQ 12 mo<br>DAS28 12 mo
Morgan, 2015	UK	255	55.92 (12.27)	21.9		HAQ 12 mo<br>DAS28 12 mo<br>SF-36 12 mo
Pappas, 2017	USA	429	53.9 (11.8)	26.9	csDMARDs	HAQ 12/24 mo
Harigai, 2018	Japan	509	59.5 (13.4)	18.1	csDMARDs	HAQ 12 mo<br>DAS28 12/24 mo
Pavelka, 2018	Czech Rep.	951	51.2 (12)	19.3		HAQ 12 mo<br>DAS28 12 mo<br>SF-36 12 mo
Tanaka, 2018	Japan	173	54.3 (13.9)	27	MTX	HAQ 12 mo<br>DAS28 12 mo
Behrens, 2020	Germany	783	47.9 (9.1)	27.7	MTX	HAQ 12/24 mo<br>DAS28 12/24 mo
Dos Santos, 2020	Brazil	73	51.75 (12.36)	19.64	MTX, LEF	HAQ 12 mo<br>EQ5D 12 mo
Santos-Moreno, 2020	Columbia	109	56*	12.04	MTX	HAQ 12/24 mo<br>DAS28 12/24 mo

Table S5. Baseline characteristics of the 11 eligible longitudinal cohort studies of RA patients. *Median age. Authors:[9, 10, 11, 12, 13, 14, 15, 16, 17, 18, 19]

	Phase III RCTs (4)	Prospective Cohort Studies (11)
12 Month HAQ	4	11
24 Month HAQ	1	3
12 Month DAS28	2	9
24 Month DAS28	-	3
12 Month SF36/SF12	2	2
24 Month SF36/SF12	1	-
12 Month EQ5D	1	1

Table S6. Summary table of outcome measures recorded in selected RCTs and longitudinal cohort studies of RA patients treated with adalimumab.

	2011	2012	2013	2014	2015	2016	2017	2018	2019	2020	Total
**(a) Melanoma**											
Ipilimumab	-	-	-	303	309	258	17	27	27	27	967
Nivolumab	-	-	-	-	-	10	17	7	7	7	47
Ipi-Nivo	-	-	-	-	-	33	100	101	102	102	438
Pembrolizumab	-	-	-	13	16	33	201	202	203	204	872
**(b) RA**											
Adalimumab	7869	7926	7981	8048	8118	8189	8241	8294	8340	8379	81,387

Table S7. Estimated number of new patients starting treatment since the Mabs became available in NHS England until 2020.

	Lifetime QALYs per patient	Lifetime mAbs Cost per patient	Total Patients	Total Monetary benefit	Net Monetary benefit
**Patients from first availability of Mabs to 2020**					
**(a) Melanoma**					
Ipilimumab	3.21	76,883	967	46,424,336	- 25,597,604
Nivolumab	5.22	42,833	47	6,985,255	5,083,681
Ipi-Nivo	5.49	138,489	438	70,883,839	11,239,145
Pembrolizumab	4.35	119,096	872	91,357,995	- 10,394,459
Dacarbazine	2.25	2,433	NA	NA	NA
			2,325	215,615,425	- 19,669,237
**(b) RA**					
Adalimumab	16.33	32,410	81,387	7,443,284,409	1,819,463,072
- Pre-2019				5,914,207,826	810,956,449
- Post-2019				1,529,076,584	1,008,506,624
Methotrexate	12.67	1,275	NA	NA	NA
**Patients from 2017 to 2020**					
**(a) Melanoma**					
Ipilimumab	3.21	76,883	98	4,700,268	- 2,591,649
Nivolumab	5.22	42,833	37	5,495,814	3,999,705
Ipi-Nivo	5.49	138,489	405	65,474,426	10,381,444
Pembrolizumab	4.35	119,096	810	84,828,833	- 9,651,589
Dacarbazine	2.25	2,433	NA	NA	NA
			1,350	160,499,340	- 2,137,910
**(b) RA**					
Adalimumab	16.33	32,410	33,255	3,041,344,261	1,215,868,837
- Pre-2019				1,512,267,677	207,362,213
- Post-2019				1,529,076,584	1,008,506,624
Methotrexate	12.67	1,275	NA	NA	NA

Table S8. Estimated lifetime QALYs, cost (£) of Mabs, total patients, total monetary benefit, and net monetary benefit, in NHS England

	Input Variables		Net Monetary benefit		Threshold Value
	Lower Limit	Upper Limit	Input Var. at Lower Limit	Input Var. at Upper Limit	For Net Return to Turn 0
**(a) Melanoma**					
**Cost Discount**					
Ipilimumab	0.00	0.99	- 18,342,204	39,700,999	0.35
Nivolumab	0.00	0.99	5,254,709	6,622,938	-2.97
Ipi-Nivo	0.00	0.99	16,835,571	61,606,974	-0.20
Pembrolizumab	0.00	0.99	- 860,366	75,412,379	0.11
**Hazard ratio mortality (vs Nivolumab)**					
Ipilimumab	1.25	1.89	9,530,779	- 53,637,887	1.32
Ipi-Nivo	0.63	1.05	49,772,875	- 16,951,680	0.90
Pembrolizumab	0.76	1.44	65,853,280	- 64,617,277	1.00
**Baseline probability (probability of mortality of Nivolumab)**					
Nivolumab	0.08	0.23	11,009,400	3,061,262	0.18
**Utility baseline**					
Ipilimumab	0.36	0.90	-78,189,094	- 12,913,200	1.01
Nivolumab	0.36	0.90	561,495	6,392,904	0.30
Ipi-Nivo	0.26	0.90	- 38,043,950	37,688,246	0.58
Pembrolizumab	0.23	0.90	- 78,618,102	30,269,071	0.71

**Utility change (vs baseline)**					
Ipilimumab	- 0.05	0.01	- 28,227,178	- 22,968,030	0.18
Nivolumab	- 0.00	0.08	4,641,979	5,525,382	-0.43
Ipi-Nivo	- 0.47	0.43	-42,228,364	64,706,655	-0.11
Pembrolizumab	0.01	0.02	- 11,614,365	- 9,174,553	0.08
**Willingness to pay**					
Ipilimumab	20,000	80,000	- 53,452,206	2,256,998	77570.00
Nivolumab	20,000	80,000	892,527	9,274,834	13612.00
Ipi-Nivo	20,000	80,000	- 31,291,158	53,769,449	42073.00
Pembrolizumab	20,000	80,000	- 65,209,256	44,420,338	55689.00
**(b) Rheumatoid arthritis**					
**Cost Discount**					
Adalimumab (pre-2019)	- 0.10	0.10	2,510,508,310	3,555,879,383	- 0.58
Adalimumab (post-2019)	- 0.10	0.10	2,985,682,492	3,080,705,201	- 6.38
**HAQ**					
Adalimumab	0.56	1.03	4,110,003,591	1,819,463,073	1.36
**Utility**					
Discontinued Adalimumab	0.31	0.92	- 8,726,553,715	14,792,941,408	0.54
**Probability Discontinued Adalimumab**	0.05	0.15	- 470,702,842	4,717,697,098	0.06
**Willingness to pay**					
Adalimumab	20,000	30,000	1,301,790,810	4,764,596,883	16,241

Table S9. One-way sensitivity analyses to examine how the net monetary values (£) changed with the lower and the upper limits of input variables, with the threshold values at which the net monetary benefit turned 0.

Appendices

Appendix 1. Database details

For the melanoma and RA meta-analyses, two reviewers independently searched the MEDLINE, Embase and Cochrane databases (from database inception to June 2021) to extract eligible studies, and a third reviewer resolved any queries. Excluded from both searches were conference abstracts, case reports, letters to the editor, review articles, and case-control studies. Management of all potentially eligible studies identified in the database search and downloaded used the data management programme Rayyan. Two reviewers independently assessed risk of bias on all eligible studies using the Cochrane Risk of Bias (ROB2) tool[43] for RCTs and the Newcastle Ottawa Score (NOS) for observational cohort studies. [8] Studies judged to be at high risk of bias were excluded from further analysis.

For melanoma, the search used the terms “immune checkpoint inhibitors”, “pembrolizumab” or “nivolumab" or “ipilimumab” and “melanoma” and “cohort” or “registry” or “RCT” or “randomised” or “real-world”. Among 4882 articles identified, nine RCTs reported OS data

and 13 RWD studies reported median OS and PFS. RCTs compared nivolumab, or ipilimumab (both at 3mg/kg and 10mg/kg doses), or pembrolizumab (two-weekly and three-weekly schedules), or nivolumab plus ipilimumab (ipi-nivo) with the historical chemotherapy regimen (dacarbazine). Nivolumab was designated the reference treatment for statistical comparisons.

The RA search identified 5348 articles, of which four Phase III RCTs and 11 observational cohort studies met the inclusion criteria (Figure 2). Of these four RCTs, three were judged to have a low risk of bias and one was considered to have some sources of bias (Supplemental Fig S4). Of the 11 included cohort studies, five were judged to have minimal risk of bias, while six had some sources of potential bias (Supplemental Fig S5).

For RA, details of the selected RCT (n=4) and cohort studies (n=11) and their baseline characteristics are shown in Supplemental Tables ST3 and ST4. One of these RCTs[6] used two different dosing regimens of adalimumab (40mg and 20mg fortnightly, respectively), but only the 40mg-fortnightly dosing group remained in our analyses because this used the licenced dose of adalimumab for treating RA. The RCTs recruited participants from the USA and Canada (n=2), Europe (n=3), and Australia (n=1). Of the observational cohort studies, all but one recruited from single areas: Europe (n=5); Japan (n=2); North America (n=2); South America (n=2). For the RCTs, the mean age of patients ranged from 47 to 56 years; the proportion of male patients ranged from 24% to 42%. In the cohort studies the mean age ranged from 48 to 60 years; the proportion of males ranged from 12% to 30%.

Reporting of HAQ-DI at 12 months was complete for the selected RCTs (n=4) and prospective cohort studies (n=11). Reporting of other outcome measures was less complete (see Supplemental Tables ST6 and ST7).

Appendix 2. Summary of data sources for input variables

Input variables were identified through the SLR by specialist clinicians in our team. In the absence of appropriate data from the SLR or further literature searches to identify relevant data, prioritising those from multinational studies or studies conducted in the UK/England for higher generalisability.

Input Variables	Melanoma	Rheumatoid Arthritis
Annual cost of Mabs	• British National Formulary List Price	• NHS England Reference Price
Utility values	• EQ-5D utility values in studies[44, 45, 46] identified by our systematic review (small number of studies; unable to meta-analyse)<br>• Economic evaluation in England[47]	• HAQ values meta-analysed by our systematic review, converted to utility values using Birmingham Rheumatoid Arthritis Model mapping equation[48]
Mortality	• Hazard ratios of mortality for each mab relative to nivolumab meta-analysed by our systematic review.<br>• Multi-national longitudinal study[20, 21, 49]	• Multi-national longitudinal study[50]
Probability of discontinuing from Mabs	(Not applicable)	• Multi-national longitudinal study[50]
Patient population	• Estimated based on prevalence and	• Estimated based on prevalence and

size	incidence rate reported in published literature, accounting for the proportion of patients fulfilling treatment criteria and receiving treatment (see Appendix 4)	incidence rate reported in published literature, accounting for the proportion of patients fulfilling treatment criteria and receiving treatment (see Appendix 4)
Willingness to pay	● NICE willingness-to-pay threshold for end-of-life[51]	● NICE willingness-to-pay threshold[52]

Input 1: Annual cost of the Mab regimens and their respective comparators. For melanoma, the calculation of cost was based on the British-National-Formulary-listed price.[53, 54, 55, 56] For RA, two costs of adalimumab were used: cost before biosimilars were available (before 2019), calculated from the BNF-listed price; [57] and cost from 2019 based on the NHS-England-listed price.[58] The cost of methotrexate was based on the BNF-listed price.[59] The calculations of total costs assumed no discount and accounted for the dose, frequency, and duration recommended in the UK (Appendix 5).[60, 61, 62, 63, 64, 65] The four Mab regimens for melanoma were administered to patients in the pre-progressed state for a specific duration, whereas adalimumab was administered to patients in the treated-with-adalimumab state perpetually. Costs included administration of the Mabs, based on NHS reference prices; this is incurred by all melanoma Mab administrations, but only by adalimumab in year-1 because patients may self-administer adalimumab at home after year-1. Where appropriate, costs were inflated to year-2021 using the UK consumer price index for health.[66]

Input 2: Utility value (UV) for each health state. In the melanoma model, for pre-progressed health state, UVs were based on published studies[44, 45, 46] identified through our SLR; for the post-progressed health state, UV, also used in an economic evaluation of checkpoint inhibitors in England, originated from a multi-national RCT.[47, 67] In the RA model, UV for the adalimumab health state was converted (using RA model mapping equation (Equation 1)[48] – see Equation 1) from Health Assessment Questionnaire – Disability Index (HAQ-DI) score derived by meta-analysis; the discontinued-from-adalimumab state shared the same UV, based on the conservative assumption that patients who discontinued from adalimumab will benefit similarly from other Mabs not investigated in this study. In both melanoma and RA models, the death state has a UV of 0. QALYs are calculated by multiplying UVs by the duration a patient stays within respective health states and summed for all health states.

$$\text{Utility value} = 0.804 - 0.203 \times \text{HAQ-DI} - 0.045 \times (\text{HAD-DI})^2 \quad \textbf{Eq. 1}$$

Input 3: Mortality. For melanoma, the mortality HRs for each Mab regimen relative to nivolumab was estimated by our SLR; the probability of death for nivolumab or the probabilities of transitioning to post-progression state for all Mabs were obtained from follow-up studies of multi-national RCTs.[20, 21, 49] For RA, the probability of death followed that of England's life table, based on the assumption that RA has no effect on mortality.

Input 4: Probability of discontinuation from Mabs. Only required for the RA model of discontinued-from-adalimumab state, this was obtained from a multi-national longitudinal study.[50] Melanoma patients in palliative care are only treated with a single Mab rather than

transitioning to a new Mab (Appendix 5).

Input 5: Patient-population size in England. Of several sources of historical data explored, none was suitable within the timeframe of our study (Appendix 6). Our estimate of the patient-population size in England was therefore based on the reported prevalence and incidence of melanoma and RA, accounting for the proportion of patients fulfilling treatment criteria and receiving treatment (Appendix 5).

Input 6: Willingness to pay (WTP) per QALY gained. For Stages 3 and 4 melanomas fulfilling the National Institutes of Health & Care Excellence (NICE) end-of-life criteria, the model adopted a WTP of £50,000[51] at base-case, with £20,000 and £80,000 for sensitivity analyses. The RA model adopted a WTP of £25,000 at base-case, with £20,000 and £30,000 for sensitivity analyses.

Analytical approach. Base-case analyses employed the total and net monetary benefits on QALYs estimated for each Mab treatment for all patients starting treatment when the Mab became available in NHS England, using the base-case (mean) values of all data. The *total* monetary benefit for each Mab was estimated with Equation 2. The *net* monetary benefit for each Mab is estimated with Equation 3. A positive net monetary benefit would suggest that the Mab is potentially cost-effective at the specified level of WTP; a negative net monetary benefit suggests that the Mab may not be cost-effective, assuming the source-information is accurate.

Total monetary benefit =QALY of mAb-QALY of comparator ×WTP per QALY	**Eq. 2**
Net monetary benefit =Total monetary benefit -(Cost of mAb- Cost of comparator)	**Eq. 3**

Appendix 3. Input variables point estimates and ranges

Input Variables	Base-case	Lower Limit	Upper Limit	References
**(a) Melanoma**				
**Utility (Baseline)**				
Dacarbazine	0.71	0.31	0.90	45
Ipilimumab	0.80	0.22	0.90	46
Nivolumab	0.78	0.22	0.90	45
Ipi-Nivo	0.68	0.22	0.90	44
Pembrolizumab	0.65	0.21	0.90	44
**Utility (Change)**				
Dacarbazine	0.03	- 0.11	0.04	45
Ipilimumab	- 0.03	- 0.05	- 0.01	46
Nivolumab	0.04	- 0.00	0.08	45
Ipi-Nivo	- 0.02	- 0.47	0.43	46
Pembrolizumab	0.15	0.01	0.02	44
**Utility**				

Post-progression	0.73	-	-	67
**Annual Probabilities**				
Progressed (Dacarbazine)	0.69	-	-	49
Progressed (Ipilimumab)	0.40	-	-	21
Progressed (Nivolumab)	0.22	-	-	21
Progressed (Ipi-Nivo)	0.19	-	-	21
Progressed (Pembrolizumab)	0.27	-	-	21
Mortality (Nivolumab)	0.15	0.08	0.23	21
**Hazard Ratio of Mortality**				
Dacarbazine	2.15	1.87	2.47	Meta-analysis
Ipilimumab	1.54	1.25	1.89	Meta-analysis
Ipilimumab + Nivolumab	0.82	0.63	1.05	Meta-analysis
Pembrolizumab	1.05	0.76	1.44	Meta-analysis
**Annual Cost of mAbs / Comparators**				
Dacarbazine[1]	550	-	-	55
Ipilimumab[2]	75,000	-	-	56
Nivolumab[3]	36,335	-	-	53
Ipi-Nivo[4]	127,660	-	-	53, 56
Pembrolizumab (1st year)[5]	84,160	-	-	54
Pembrolizumab (2nd year)[5]	34,365	-	-	54
Discount	0	0	0.99	Assumption for threshold analysis
**Cost of Administration**				
First dose (HRG SB 13Z)	471	-	-	68
Subsequent (HRG SB 15Z)	481	-	-	68
**WTP per QALY**	50000	20000	80000	51
**(b) Rheumatoid arthritis**				
**HAQ-DI**				
Adalimumab	0.79	0.56	1.03	Meta-analysis
Methotrexate	1.31	0.99	1.63	Meta-analysis
**Utility**				
Discontinued adalimumab	0.62	0.31	0.92	Converted from HAQ-DI for adalimumab; assumed same as adalimumab.
**Annual Probabilities**				
Discontinuation of adalimumab	0.10	0.05	0.15	50
Death	0.0042	0.0021	0.0063	50
**Annual Cost of mAbs / Comparators**				
Adalimumab (post-2019)[6]	3679	-	-	58
Adalimumab (pre-2019)[6]	9156	-	-	57
Methotrexate[7]	41	-	-	59
Discount for adalimumab	0	0	0.99	Assumption for threshold analysis

**Cost of Administration**				
First year adalimumab	134	-	-	[48]
First year monitoring cost of adalimumab	180	-	-	[48]
Ongoing monitoring cost of methotrexate	135	-	-	[48]
**WTP per QALY**	25000	20000	30000	[52]

1. *Dacarbazine 250mg/m2 5 days every 3 weeks, until progression*
2. *Ipilimumab 3mg/kg for 4 cycles*
3. *Nivolumab 240mg every 2 weeks, until progression*
4. *Ipilimumab 3mg/kg for 4 cycles, followed by nivolumab 240mg every 2 weeks, until progression*
5. *Pembrolizumab 100mg every 3 weeks, until progression. Patients will only incur the second-year cost if they survive the first year within the pre-progressed health state.*
6. *Adalimumab 40mg / ml every 2 weeks before and after biosimilars are available in England NHS in year 2019*
7. *Methotrexate 20mg weekly*

Appendix 4. Calculations of patient population size in England for (A) Stages 3 or 4 unresectable melanoma and (B) RA

	2011	2012	2013	2014	2015	2016	2017	2018	2019	2020	Ref.
**(a) Melanoma**											
England Population ('000)	NA	NA	NA	54,316.6	54,786.3	55,268.1	55,619.4	55,977.2	56,287.0	56,550.0	[66]
Patients Living with Melanoma, a	NA	NA	NA	127,833	138,250	148,856	159,653	170,259	180,758	191,141	[66]
Crude incidence (per 100,000)	NA	NA	NA	23.90	24.40	24.90	24.75	24.75	24.75	24.75	[69]
New Diagnoses, b	NA	NA	NA	12,951	13,334	13,725	13,726	13,812	13,886	13,949	Calculated
Total Melanoma Patients, a + b	NA	NA	NA	140,784	151,584	162,580	173,380	184,072	194,645	205,090	Calculated
Survival Rate	NA	NA	NA	0.98	0.98	0.98	0.98	0.98	0.98	0.98	[70]
Total Patients Survived 1 Year	NA	NA	NA	138,250	148,856	159,654	170,259	180,758	191,141	201,398	Calculated
% Stages 3 or 4	NA	NA	NA	0.09	0.09	0.09	0.09	0.09	0.09	0.09	[71]
Patients with Stages 3 or 4	NA	NA	NA	12,857	13,844	14,848	15,834	16,811	17,776	18,730	Calculated
% Treated	NA	NA	NA	0.06	0.06	0.06	0.06	0.06	0.06	0.06	[28]
% Treated with Selected Mabbs as First Line	NA	NA	NA	0.42	0.42	0.42	0.42	0.42	0.42	0.42	
Total Patients Starting Treatment	NA	NA	NA	**316**	**325**	**335**	335	337	338	340	Calculated
% Ipilimumab	NA	NA	NA	0.96	0.95	0.77	0.05	0.08	0.08	0.08	[28]
% Nivolumab	NA	NA	NA	0	0	0.03	0.05	0.02	0.02	0.02	
% Ipi-Nivo	NA	NA	NA	0	0	0.1	0.3	0.3	0.3	0.3	
% Pembrolizumab	NA	NA	NA	0.04	0.05	0.1	0.6	0.6	0.6	0.6	
N Ipilimumab	NA	NA	NA	**303**	**309**	**258**	17	27	27	27	Calculated
N Nivolumab	NA	NA	NA	**0**	**0**	**10**	17	7	7	7	Calculated
N Ipi-Nivo	NA	NA	NA	**0**	**0**	**33**	100	101	102	102	Calculated
N Pembro-	NA	NA	NA	**13**	**16**	**33**	201	202	203	204	Calculated

lizumab

**(b) RA**											
England Population	53,107.2	53,493.7	53,865.8	54,316.6	54,786.3	55,268.1	55,619.4	55,977.2	56,287.0	56,550.0	[66]
Crude prevalence (per 100,000)	0.004877	0.004877	0.004877	0.004877	0.004877	0.004877	0.004877	0.004877	0.004877	0.004877	[72]
Individuals Living with RA, a	259025	260910	262725	264924	267215	269565	271278	273023	274534	275817	Calculated
Crude incidence (per 100,000)	0.000410	0.000410	0.000410	0.000410	0.000410	0.000410	0.000410	0.000410	0.000410	0.000410	[72]
New Diagnoses, b	21673	21831	21983	22167	22358	22554	22698	22844	22971	23078	Calculated
Total RA Patients, a + b	280698	282741	284708	287090	289572	292120	293976	295867	297505	298895	Calculated
Survival Rate	0.996761	0.996761	0.996761	0.996761	0.996761	0.996761	0.996761	0.996761	0.996761	0.996761	[73]England life table (50 y.o.)
Total Patients Survived 1 Year	279,789	281,825	283,786	286,160	288,635	291,173	293,024	294,909	296,541	297,927	Calculated
% Met Treatment Criteria[1]	0.0625	0.0625	0.0625	0.0625	0.0625	0.0625	0.0625	0.0625	0.0625	0.0625	[74]
Total Met Treatment Criteria[1]	17,487	17,614	17,737	17,885	18,040	18,198	18,314	18,431	18,534	18,620	Calculated
% Treated with Adalimumab	0.45	0.45	0.45	0.45	0.45	0.45	0.45	0.45	0.45	0.45	[75]
Total Treated with Adalimumab	7869	7926	7981	8048	8118	8189	8241	8294	8340	8379	Calculated

1. Patients with moderate RA (DAS28 between 3.2 and 5.1) in whom intensive therapy with ≥2 conventional disease modifying anti-rheumatic drugs (csDMARDs) has not controlled the disease well enough

Appendix 5. Dose, frequency, and duration of administration assumed in calculating the cost of the Mabs

	Dose	Frequency	Duration	References
**Melanoma**				
Ipilimumab	3mg / kg	4 times	-	[44, 62]
Nivolumab	240mg	Every 2 weeks	Until progression (6.9 months)	[44, 62, 63]
Ipi-Nivo	3mg / kg ipilimumab, with 70mg nivolumab then 240mg nivolumab	Ipilimumab 4 times, then nivolumab every 2 weeks	Until progression (11.5 months)	[62, 63]
Pembrolizumab	200mg	Every 3 weeks	Until progression (16.9 months)	[21, 65]
Dacarbazine	250mg / m^2 body surface area (1.79m^2)	5 days every 3 weeks	Until progression (2.2 months)	[36, 60]
**Rheumatoid arthritis**				
Adalimumab	40mg	Every 2 weeks	Perpetual	[61]
Methotrexate	20mg	Weekly	Perpetual	[64]

Appendix 6. List of historical data sources explored for population size

Aggregate data for the systemic anti-cancer therapy (SACT) dataset. This dataset did not indicate the type of cancer and whether the Mabs were used as the first-line treatment.
OPENSafely analytic platform. This platform was only available for COVID-19 research at the time of this study.
The British Society for Rheumatology Biologics Register which tracks the progress of RA patients prescribed a biologic (including biosimilars. This registry is voluntary hence would not give a reliable patient population size in England.

References

1. Gorry C, McCullagh L, Barry M. Economic Evaluation of Systemic Treatments for Advanced Melanoma: A Systematic Review. Value Health. 2020;23(1):52-60.
2. Chen YF, Jobanputra P, Barton P, Jowett S, Bryan S, Clark W, et al. A systematic review of the effectiveness of adalimumab, etanercept and infliximab for the treatment of rheumatoid arthritis in adults and an economic evaluation of their cost-effectiveness. Health Technol Assess. 2006;10(42):iii-iv, xi-xiii, 1-229.
3. Sterne JAC, Savović J, Page MJ, Elbers RG, Blencowe NS, Boutron I, et al. RoB 2: a revised tool for assessing risk of bias in randomised trials. Bmj. 2019;366:l4898.
4. Bejarano V, Quinn M, Conaghan PG, Reece R, Keenan AM, Walker D, et al. Effect of the early use of the anti-tumor necrosis factor adalimumab on the prevention of job loss in patients with early rheumatoid arthritis. Arthritis Rheum. 2008;59(10):1467-74.
5. Hørslev-Petersen K, Hetland ML, Junker P, Pødenphant J, Ellingsen T, Ahlquist P, et al. Adalimumab added to a treat-to-target strategy with methotrexate and intra-articular triamcinolone in early rheumatoid arthritis increased remission rates, function and quality of life. The OPERA Study: an investigator-initiated, randomised, double-blind, parallel-group, placebo-controlled trial. Ann Rheum Dis. 2014;73(4):654-61.
6. Keystone EC, Kavanaugh AF, Sharp JT, Tannenbaum H, Hua Y, Teoh LS, et al. Radiographic, clinical, and functional outcomes of treatment with adalimumab (a human anti-tumor necrosis factor monoclonal antibody) in patients with active rheumatoid arthritis receiving concomitant methotrexate therapy: a randomized, placebo-controlled, 52-week trial. Arthritis Rheum. 2004;50(5):1400-11.
7. Strand V, Rentz AM, Cifaldi MA, Chen N, Roy S, Revicki D. Health-related quality of life outcomes of adalimumab for patients with early rheumatoid arthritis: results from a randomized multicenter study. J Rheumatol. 2012;39(1):63-72.
8. Stang A. Critical evaluation of the Newcastle-Ottawa scale for the assessment of the quality of nonrandomized studies in meta-analyses. Eur J Epidemiol. 2010;25(9):603-5.
9. Behrens F, Tony HP, Koehm M, Schwaneck EC, Gnann H, Greger G, et al. Sustained improvement in work outcomes in employed patients with rheumatoid arthritis during 2 years of adalimumab therapy: an observational cohort study. Clin Rheumatol. 2020;39(9):2583-92.
10. Burmester GR, Matucci-Cerinic M, Mariette X, Navarro-Blasco F, Kary S, Unnebrink K, et al. Safety and effectiveness of adalimumab in patients with rheumatoid arthritis over 5 years of therapy in a phase 3b and subsequent postmarketing observational study. Arthritis Res Ther. 2014;16(1):R24.
11. Dos Santos JBR, da Silva MRR, Kakehasi AM, Acurcio FA, Almeida AM, Alves de Oliveira Junior H, et al. First line of subcutaneous anti-tnf therapy for rheumatoid arthritis: A prospective cohort study. Expert Rev Clin Immunol. 2020;16(12):1217-25.

12. Harigai M, Tsuchiya T, Kawana K, Kurimoto S. Long-term safety and effectiveness of adalimumab for the treatment of Japanese patients with rheumatoid arthritis: 3-year results from a postmarketing surveillance of 552 patients. Mod Rheumatol. 2018;28(1):30-8.
13. Kievit W, Adang EM, Fransen J, Kuper HH, van de Laar MA, Jansen TL, et al. The effectiveness and medication costs of three anti-tumour necrosis factor alpha agents in the treatment of rheumatoid arthritis from prospective clinical practice data. Ann Rheum Dis. 2008;67(9):1229-34.
14. Morgan C, McBeth J, Cordingley L, Watson K, Hyrich KL, Symmons DP, et al. The influence of behavioural and psychological factors on medication adherence over time in rheumatoid arthritis patients: a study in the biologics era. Rheumatology (Oxford). 2015;54(10):1780-91.
15. Pappas DA, Kremer JM, Griffith J, Reed G, Salim B, Karki C, et al. Long-Term Effectiveness of Adalimumab in Patients with Rheumatoid Arthritis: An Observational Analysis from the Corrona Rheumatoid Arthritis Registry. Rheumatol Ther. 2017;4(2):375-89.
16. Pavelka K, Zavada J, Kristkova Z, Szczukova L. FRI0122 Drug survival of adalimumab in patients with rheumatoid arthritis, ankylosing spondylitis and psoriatic arthritis over 10 years in real-world setting of czech registry attra. Annals of the Rheumatic Diseases. 2018;77(Suppl 2):605-6.
17. Pope JE, Haraoui B, Rampakakis E, Psaradellis E, Thorne C, Sampalis JS. Treating to a target in established active rheumatoid arthritis patients receiving a tumor necrosis factor inhibitor: results from a real-world cluster-randomized adalimumab trial. Arthritis Care Res (Hoboken). 2013;65(9):1401-9.
18. Santos-Moreno P, Sánchez-Vanegas G. Clinical Effectiveness and Safety of Treatment With Anti-Tumor Necrosis Factor α Drugs in a Cohort of Colombian Patients With Rheumatoid Arthritis. J Clin Rheumatol. 2020;26(7S Suppl 2):S123-s30.
19. Tanaka Y, Mimori T, Yamanaka H, Nakajima R, Morita K, Kimura J, et al. Effectiveness and safety of initiating adalimumab plus ≥12 mg/week methotrexate with adjustable dosing in biologic-naïve patients with early rheumatoid arthritis: HAWK study postmarketing surveillance in Japan. Mod Rheumatol. 2019;29(4):572-80.
20. Hamid O, Robert C, Daud A, Hodi FS, Hwu WJ, Kefford R, et al. Five-year survival outcomes for patients with advanced melanoma treated with pembrolizumab in KEYNOTE-001. Ann Oncol. 2019;30(4):582-8.
21. Larkin J, Chiarion-Sileni V, Gonzalez R, Grob JJ, Rutkowski P, Lao CD, et al. Five-Year Survival with Combined Nivolumab and Ipilimumab in Advanced Melanoma. N Engl J Med. 2019;381(16):1535-46.
22. Gogas HJ, Kirkwood JM, Sondak VK. Chemotherapy for metastatic melanoma: time for a change? Cancer. 2007;109(3):455-64.
23. van de Putte LB, Atkins C, Malaise M, Sany J, Russell AS, van Riel PL, et al. Efficacy and safety of adalimumab as monotherapy in patients with rheumatoid arthritis for whom previous disease modifying antirheumatic drug treatment has failed. Ann Rheum Dis. 2004;63(5):508-16.
24. Weinblatt ME, Keystone EC, Furst DE, Moreland LW, Weisman MH, Birbara CA, et al. Adalimumab, a fully human anti-tumor necrosis factor alpha monoclonal antibody, for the treatment of rheumatoid arthritis in patients taking concomitant methotrexate: the ARMADA trial. Arthritis Rheum. 2003;48(1):35-45.
25. Ascierto PA, Del Vecchio M, Mackiewicz A, Robert C, Chiarion-Sileni V, Arance A, et al. Overall survival at 5 years of follow-up in a phase III trial comparing ipilimumab 10 mg/kg with 3 mg/kg in patients with advanced melanoma. J Immunother Cancer. 2020;8(1).
26. Robert C, Long GV, Brady B, Dutriaux C, Di Giacomo AM, Mortier L, et al. Five-Year Outcomes With Nivolumab in Patients With Wild-Type BRAF Advanced Melanoma. J Clin Oncol. 2020;38(33):3937-46.

27. Schachter J, Ribas A, Long GV, Arance A, Grob JJ, Mortier L, et al. Pembrolizumab versus ipilimumab for advanced melanoma: final overall survival results of a multicentre, randomised, open-label phase 3 study (KEYNOTE-006). Lancet. 2017;390(10105):1853-62.
28. Board R, Smittenaar R, Lawton S, Liu H, Juwa B, Chao D, et al. Metastatic melanoma patient outcomes since introduction of immune checkpoint inhibitors in England between 2014 and 2018. Int J Cancer. 2021;148(4):868-75.
29. Borges FC, Ramos C, Ramos A, Mendes GP, Murteira R, Soares P, et al. Monitoring real-life utilization of pembrolizumab in advanced melanoma using the Portuguese National Cancer Registry. Pharmacoepidemiol Drug Saf. 2021;30(3):342-9.
30. Casarotto E, Chandwani S, Mortier L, Dereure O, Dutriaux C, Dalac S, et al. Real-world effectiveness of pembrolizumab in advanced melanoma: analysis of a French national clinicobiological database. Immunotherapy. 2021;13(11):905-16.
31. Cowey CL, Liu FX, Black-Shinn J, Stevinson K, Boyd M, Frytak JR, et al. Pembrolizumab Utilization and Outcomes for Advanced Melanoma in US Community Oncology Practices. J Immunother. 2018;41(2):86-95.
32. Cowey CL, Scherrer E, Boyd M, Aguilar KM, Beeks A, Krepler C. Pembrolizumab Utilization and Clinical Outcomes Among Patients With Advanced Melanoma in the US Community Oncology Setting: An Updated Analysis. J Immunother. 2021;44(6):224-33.
33. Dalle S, Mortier L, Corrie P, Lotem M, Board R, Arance AM, et al. Long-term real-world experience with ipilimumab and non-ipilimumab therapies in advanced melanoma: the IMAGE study. BMC Cancer. 2021;21(1):642.
34. Hogg D, Monzon JG, Ernst S, Song X, McWhirter E, Savage KJ, et al. Canadian cohort expanded-access program of nivolumab plus ipilimumab in advanced melanoma. Curr Oncol. 2020;27(4):204-14.
35. Jochems A, Leeneman B, Franken MG, Schouwenburg MG, Aarts MJB, van Akkooi ACJ, et al. Real-world use, safety, and survival of ipilimumab in metastatic cutaneous melanoma in The Netherlands. Anticancer Drugs. 2018;29(6):572-8.
36. Liu FX, Ou W, Diede SJ, Whitman ED. Real-world experience with pembrolizumab in patients with advanced melanoma: A large retrospective observational study. Medicine (Baltimore). 2019;98(30):e16542.
37. Mohr P, Ascierto P, Arance A, McArthur G, Hernaez A, Kaskel P, et al. Real-world treatment patterns and outcomes among metastatic cutaneous melanoma patients treated with ipilimumab. J Eur Acad Dermatol Venereol. 2018;32(6):962-71.
38. Moser JC, Chen D, Hu-Lieskovan S, Grossmann KF, Patel S, Colonna SV, et al. Real-world survival of patients with advanced BRAF V600 mutated melanoma treated with front-line BRAF/MEK inhibitors, anti-PD-1 antibodies, or nivolumab/ipilimumab. Cancer Med. 2019;8(18):7637-43.
39. Moser JC, Wei G, Colonna SV, Grossmann KF, Patel S, Hyngstrom JR. Comparative-effectiveness of pembrolizumab vs. nivolumab for patients with metastatic melanoma. Acta Oncol. 2020;59(4):434-7.
40. Pavlick AC, Zhao R, Lee CH, Ritchings C, Rao S. First-line immunotherapy versus targeted therapy in patients with BRAF-mutant advanced melanoma: a real-world analysis. Future Oncol. 2021;17(6):689-99.
41. Tarhini A, Atzinger C, Gupte-Singh K, Johnson C, Macahilig C, Rao S. Treatment patterns and outcomes for patients with unresectable stage III and metastatic melanoma in the USA. J Comp Eff Res. 2019;8(7):461-73.
42. Margolin K. Effectiveness and safety of ipilimumab therapy in advanced melanoma: evidence from clinical practice sites in the US. THE JOURNAL OF COMMUNITY AND SUPPORTIVE ONCOLOGY. 2015.

43. Higgins JP, Altman DG, Gøtzsche PC, Jüni P, Moher D, Oxman AD, et al. The Cochrane Collaboration's tool for assessing risk of bias in randomised trials. Bmj. 2011;343:d5928.
44. Joseph RW, Liu FX, Shillington AC, Macahilig CP, Diede SJ, Dave V, et al. Health-related quality of life (QoL) in patients with advanced melanoma receiving immunotherapies in real-world clinical practice settings. Qual Life Res. 2020;29(10):2651-60.
45. Long GV, Atkinson V, Ascierto PA, Robert C, Hassel JC, Rutkowski P, et al. Effect of nivolumab on health-related quality of life in patients with treatment-naïve advanced melanoma: results from the phase III CheckMate 066 study. Ann Oncol. 2016;27(10):1940-6.
46. Schadendorf D, Larkin J, Wolchok J, Hodi FS, Chiarion-Sileni V, Gonzalez R, et al. Health-related quality of life results from the phase III CheckMate 067 study. Eur J Cancer. 2017;82:80-91.
47. Robert C, Long GV, Brady B, Dutriaux C, Maio M, Mortier L, et al. Nivolumab in previously untreated melanoma without BRAF mutation. N Engl J Med. 2015;372(4):320-30.
48. Malottki K, Barton P, Tsourapas A, Uthman AO, Liu Z, Routh K, et al. Adalimumab, etanercept, infliximab, rituximab and abatacept for the treatment of rheumatoid arthritis after the failure of a tumour necrosis factor inhibitor: a systematic review and economic evaluation. Health Technol Assess. 2011;15(14):1-278.
49. Ascierto PA, Long GV, Robert C, Brady B, Dutriaux C, Di Giacomo AM, et al. Survival Outcomes in Patients With Previously Untreated BRAF Wild-Type Advanced Melanoma Treated With Nivolumab Therapy: Three-Year Follow-up of a Randomized Phase 3 Trial. JAMA Oncol. 2019;5(2):187-94.
50. Furst DE, Kavanaugh A, Florentinus S, Kupper H, Karunaratne M, Birbara CA. Final 10-year effectiveness and safety results from study DE020: adalimumab treatment in patients with rheumatoid arthritis and an inadequate response to standard therapy. Rheumatology (Oxford). 2015;54(12):2188-97.
51. Bovenberg J, Penton H, Buyukkaramikli N. 10 Years of End-of-Life Criteria in the United Kingdom. Value Health. 2021;24(5):691-8.
52. McCabe C, Claxton K, Culyer AJ. The NICE cost-effectiveness threshold: what it is and what that means. Pharmacoeconomics. 2008;26(9):733-44.
53. British National Formulary (BNF). Nivolumab Medicinal Forms London, UK: National Institute for Health & Care Excellence; 2022 [updated 31/08/2022. Available from: https://bnf.nice.org.uk/drugs/nivolumab/.
54. British National Formulary (BNF). Pembrolizumab Medicinal Forms London, UK: National institute for Health & Care Excellence; 2022 [updated 31/08/2022. Available from: https://bnf.nice.org.uk/drugs/pembrolizumab/.
55. British National Formulary (BNF). Dacarbazine Medicinal forms London, UK: National institute for Health & Care Excellence; 2022 [updated 31/08/2022. Available from: https://bnf.nice.org.uk/drugs/dacarbazine/medicinal-forms/.
56. British National Formulary (BNF). Ipilimumab Medicinal Forms London, UK: https://bnf.nice.org.uk/drugs/ipilimumab/; 2022 [updated 31/08/2022. Available from: https://bnf.nice.org.uk/drugs/ipilimumab/.
57. British National Formulary (BNF). Adalimumab Medicinal Forms 2022 [Available from: https://bnf.nice.org.uk/drugs/adalimumab/medicinal-forms/.
58. Swindells M. Reference Prices for Adalimumab. In: Improvement NEaN, editor. London, UK2019.

59. British National Formulary (BNF). Methotrexate Medicinal Forms London, UK: National Institute for Health & Care Excellence; 2022 [updated 31/08/2022. Available from: https://bnf.nice.org.uk/drugs/methotrexate/.
60. Electronic Medical Compendium. Dacarbazine 100 mg powder for solution for injection/infusion UK: Datapharm; 2020 [updated 20/11/2020.
61. Electronic Medical Compendium. Humira 40 mg/0.4 ml solution for injection in pre-filled pen UK: Datapharm; 2021 [updated 09/06/2021. Available from: https://www.medicines.org.uk/emc/product/7986/smpc.
62. Electronic Medical Compendium. YERVOY 5 mg/ml concentrate for solution for infusion UK: Datapharm; 2022 [updated 16/05/2022. Available from: https://www.medicines.org.uk/emc/medicine/24779.
63. Electronic Medical Compendium. OPDIVO 10 mg/mL concentrate for solution for infusion UK: Datapharm; 2022 [updated 18/08/2022. Available from: https://www.medicines.org.uk/emc/medicine/30476.
64. Electronic Medical Compendium. Methotrexate 2.5 mg Tablets UK: Datapharm; 2022 [updated 23/01/2020. Available from: https://www.medicines.org.uk/emc/product/511/smpc.
65. Electronic Medical Compendium. KEYTRUDA 25 mg/mL concentrate for solution for infusion UK: Datapharm; 2022 [updated 28/07/2022. Available from: https://www.medicines.org.uk/emc/product/2498/smpc.
66. Office for National Statistics. CPI INDEX 06 : HEALTH 2015=100 UK: Office for National Statistics,; 2022 [updated 14/09/2022. Available from: https://www.ons.gov.uk/economy/inflationandpriceindices/timeseries/d7bz/mm23.
67. Meng Y, Hertel N, Ellis J, Morais E, Johnson H, Philips Z, et al. The cost-effectiveness of nivolumab monotherapy for the treatment of advanced melanoma patients in England. Eur J Health Econ. 2018;19(8):1163-72.
68. NHS England. 2020/21 National Cost Collection Data Publication. 2022.
69. (IARC) IAfRoC. Crude rate per 100 000, incidence, males and females Lyon, France: International Agency for Research on Cancer 2022 2022.
70. UK. CR. Melanoma skin cancer statistics. 2022.
71. UK. CR. Early Diagnosis Data Hub UK: Cancer Research UK. 2022.
72. Gardiner J, Su B, Adomaviciute S, Watt H, Newson R, Foley K, et al. Rheumatoid arthritis prevalence models for small populations: Technical Document produced for Arthritis Research UK. London, UK: Department Primary Care & Public Health School of Public Health, Imperial College London; 2018 October 2018.
73. Office for National Statistics. National life tables: UK Office for National Statistics. 2022.
74. Wise J. Rheumatoid arthritis: NICE recommends more treatments for patients with moderate disease. Bmj. 2021;373:n1485.
75. Emery P, Solem C, Majer I, Cappelleri JC, Tarallo M. A European chart review study on early rheumatoid arthritis treatment patterns, clinical outcomes, and healthcare utilization. Rheumatol Int. 2015;35(11):1837-49.
